# Benralizumab versus placebo for hypereosinophilic syndrome: a randomized, placebo-controlled phase 3 trial

**DOI:** 10.1038/s41591-026-04315-8

**Published:** 2026-03-31

**Authors:** Princess U. Ogbogu, Florence Roufosse, Praveen Akuthota, Piotr Kuna, Matthieu Groh, Andreas Reiter, Akira Yokota, Salman H. Siddiqui, Pim G. N. J. Mutsaers, Bing Li, Paneez Khoury, Lila M. Bahadori, Artur Bednarczyk, Gerben Bouma, Laura G. Brooks, Jorge Ferreira, Hanna Grindebacke, Calvin N. Ho, Priya Jain, Rebecca L. Palmer, Maria L. Jison, Amy D. Klion

**Affiliations:** 1 Division of Pediatric Allergy, Immunology, and Rheumatology, Department of Pediatrics, University Hospitals Rainbow Babies and Children’s Hospital, Case Western Reserve University School of Medicine, Cleveland, OH USA; 2 Department of Internal Medicine, Hôpital Universitaire de Bruxelles – Site Erasme, Université Libre de Bruxelles, Brussels, Belgium; 3 Division of Pulmonary, Critical Care, Sleep Medicine and Physiology, Department of Medicine, University of California San Diego, La Jolla, CA USA; 4 Division of Internal Medicine Asthma and Allergy, Medical University of Lodz, Lodz, Poland; 5 Universtié de Versailles Saint-Quentin-en-Yvelines, Montigny-Le-Bretonneux, France; National Referral Center for Hypereosinophilic Syndromes, Department of Internal Medicine, Clinical Immunology and Hematology, Hôpital Foch, Suresnes, France; 6 Department of Hematology and Oncology, University Hospital Mannheim, Heidelberg University, Mannheim, Germany; 7 Department of Hematology, Chiba Aoba Municipal Hospital, Chiba, Japan; 8 NIHR Imperial Biomedical Research Centre, National Heart and Lung Institute, Royal Brompton and Hammersmith Hospitals, Imperial College London, London, UK; 9 Department of Hematology, Erasmus University Medical Center, Rotterdam, the Netherlands; 10 State Key Laboratory of Experimental Hematology, National Clinical Research Center for Blood Diseases, Haihe Laboratory of Cell Ecosystem, Institute of Hematology and Blood Diseases Hospital, Chinese Academy of Medical Sciences and Peking Union Medical College, Tianjin, China; 11 MDS and MPN Center, Institute of Hematology and Blood Diseases Hospital, Chinese Academy of Medical Sciences and Peking Union Medical College, Tianjin, China; 12 Laboratory of Parasitic Diseases, National Institute of Allergy and Infectious Diseases, National Institutes of Health, Bethesda, MD USA; 13 Respiratory and Immunology, BioPharmaceuticals R&D, AstraZeneca, Gaithersburg, MD USA; 14 Respiratory and Immunology, BioPharmaceuticals R&D, AstraZeneca, Warsaw, Poland; 15 Translational Science and Experimental Medicine, Respiratory and Immunology, BioPharmaceuticals R&D, AstraZeneca, Cambridge, UK; 16 Respiratory and Immunology, BioPharmaceuticals R&D, AstraZeneca, Cambridge, UK; 17 Respiratory and Immunology, BioPharmaceuticals R&D, AstraZeneca, Gothenburg, Sweden; 18 Respiratory and Immunology, BioPharmaceuticals Medical, AstraZeneca, Cambridge, UK

**Keywords:** Immunology, Medical research

## Abstract

Benralizumab, an eosinophil-depleting anti-IL-5 receptor α antibody, has demonstrated efficacy in severe eosinophilic asthma and eosinophilic granulomatosis with polyangiitis and shown promising results in hypereosinophilic syndrome (HES). NATRON was a randomized, double-blind placebo-controlled phase 3 study evaluating the efficacy and safety of benralizumab in *FIP1L1::PDGFRA*-negative HES. The primary endpoint was time to first HES flare. In total, 133 patients (median (range) age 51 (14–87) years, 62% female) were randomized (1:1) to receive benralizumab 30 mg every 4 weeks or placebo for 24 weeks, in addition to background therapy. Benralizumab significantly reduced the risk of first flare versus placebo (hazard ratio 0.35, 95% CI 0.18 to 0.69, *P* = 0.0024). Adverse events occurred in 64.2% and 66.7% of benralizumab- and placebo-treated patients, respectively. Benralizumab’s safety was consistent with its known profile. These results demonstrate the efficacy and safety of add-on benralizumab in the treatment of HES. ClinicalTrials.gov identifier: NCT04191304.

## Abstract (web summary)

Benralizumab (an anti-IL-5 receptor α antibody), compared to placebo, significantly reduced the risk of first flare in patients with hypereosinophilic syndrome.

## Main

Hypereosinophilic syndrome (HES) is defined by the presence of hypereosinophilia and evidence of eosinophil-mediated clinical manifestations^1^. Clinical presentation is highly heterogeneous, with patients experiencing symptoms of varying severity, potentially affecting multiple organ systems^2^. These features of HES, alongside rarity, often lead to substantial delays in diagnosis and treatment^2^.

Myeloid-HES, also known as primary neoplastic HES, is characterized by clonal eosinophil proliferation^1^. Myeloid-HES is associated with a wide variety of molecular abnormalities, such as gene rearrangements involving tyrosine kinase receptors (typically the *FIP1L1::PDGFRA* fusion gene)^1^. In patients with rearrangements of the *FIP1L1::PDGFRA* fusion gene, the tyrosine kinase inhibitor imatinib has shown remarkable efficacy^3^. However, treatment options for patients with imatinib-insensitive HES, including those with lymphocytic HES (an indolent T cell lymphoproliferative disease) or idiopathic HES (mechanistic cause not yet identified), are limited^4^.

Treatment for patients with nonmyeloid HES typically involves the use of corticosteroids and/or immunosuppressive or cytotoxic therapies, which can reduce inflammation and control symptoms but often lead to adverse effects and may still result in suboptimal disease control^5^. Biologic therapies have expanded treatment options in HES^4,6,7^. Results from two clinical trials demonstrated that mepolizumab, a monoclonal antibody that inhibits eosinophil activation and differentiation by binding interleukin (IL)-5^8^, reduced disease flares compared to placebo and had corticosteroid-sparing effects in patients with uncontrolled *FIP1L1::PDGFRA*-negative HES^9,10^. On the basis of these findings, mepolizumab (300 mg subcutaneous, every 4 weeks) was approved for the treatment of HES. While the approval of mepolizumab for HES has improved clinical outcomes^9^, treatment responses vary. Some patients do not achieve a clinical response, or respond only partially to mepolizumab, highlighting the need for alternative therapies^4,6,7^. A retrospective analysis of the off-label use of biologics in HES suggests that failure to respond to one biologic does not preclude a response to a different biologic^11^.

The central role of eosinophilic inflammation and eosinophil-mediated end organ damage in the pathophysiology of HES, as well as the efficacy of IL-5 inhibition with mepolizumab, suggests that a more direct eosinophil-depleting approach may also be beneficial^2^. Benralizumab is a humanized, afucosylated monoclonal antibody that binds to the α subunit of the IL-5 receptor expressed on eosinophils^12^. Benralizumab causes rapid, near-complete depletion of eosinophils in peripheral blood, bone marrow, airway sputum, skin, esophagus and gastrointestinal tissues^13–17^ via antibody-dependent cell-mediated cytotoxicity^12^. It has demonstrated clinical benefit and corticosteroid-sparing effects in severe eosinophilic asthma^18–21^ and eosinophilic granulomatosis with polyangiitis (EGPA)^22^ and is approved as an add-on maintenance treatment for patients aged 6 years and older with severe eosinophilic asthma, and for adults with EGPA. In a phase 2 study of patients with symptomatic, treatment-refractory *FIP1L1::PDGFRA*-negative HES, 90% of patients achieved a ≥50% reduction in absolute eosinophil counts (AECs) from baseline to week 12, and reduced tissue eosinophilia was observed in patients with severe disease^14^. Long-term follow-up from this study suggests that benralizumab remains effective and well tolerated^23^.

Here, we describe the results of a phase 3 trial that assessed the efficacy and safety of benralizumab compared to placebo in patients with *FIP1L1::PDGFRA*-negative HES.

## Results

### Patient disposition

**Fig. 1:**
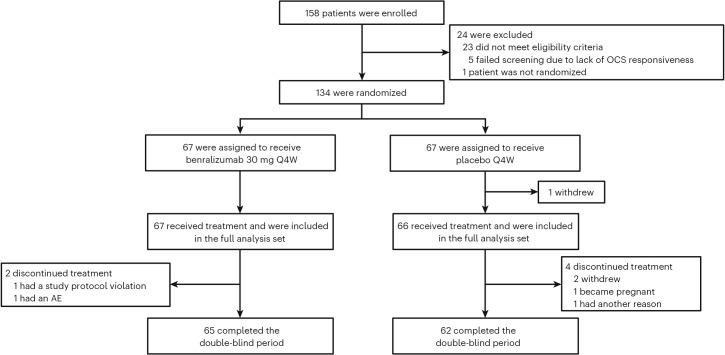
Trial profile. The flow of patients through the study, from enrollment through to completion of the double-blind period. Q4W, every 4 weeks.

The trial enrolled patients between 20 July 2020 and 13 November 2024, with the final patient visit for the double-blind period on 7 May 2025. Overall, 134 patients underwent randomization and 133 received ≥1 dose of treatment; 67 received benralizumab and 66 received placebo. In total, 97% (65/67) of benralizumab-treated patients and 92.5% (62/66) of placebo-treated patients completed the 24-week double-blind period (Fig. 1).

### Baseline demographics and disease characteristics

The demographic and clinical characteristics of the patients at baseline were generally balanced between the treatment groups and representative of the targeted patient population with HES (Table 1 and Supplementary Table 2). Overall, 62% (82/133) of patients were female, 38% (51/133) were male and the median (range) age was 51.0 (14.0–87.0) years. Four adolescents were enrolled in the study (three received benralizumab and one received placebo). Most patients (75.2%, 100/133) had idiopathic HES and 12.0% (16/133) of patients had lymphocytic HES. The median (range) time since diagnosis was 1.9 (0.1–32.3) years, and patients had experienced a median (range) of 2 (0–12) HES flares in the 12 months before enrollment. Before oral corticosteroid (OCS)-responsiveness assessment and randomization, the median (range) AEC was 1,600 (1,000–25,150) cells µl^−1^. Around three-quarters of patients (76.7%, 102/133) were receiving background systemic OCS at enrollment, at a median (range) prednisone-equivalent dose of 5.0 (0.0–30.0) mg day^−1^.

**Table 1:** Baseline characteristics

Characteristic	Benralizumab (*n* = 67)	Placebo (*n* = 66)	Total (*N* = 133)
Age (years)			
Median (range)	49.0 (14.0–87.0)	53.5 (16.0–83.0)	51.0 (14.0–87.0)
≤21	9 (13.4%)	7 (10.6%)	16 (12.0%)
22–65	45 (67.2%)	45 (68.2%)	90 (67.7%)
≥66	13 (19.4%)	14 (21.2%)	27 (20.3%)
Sex			
Female	43 (64.2%)	39 (59.1%)	82 (61.7%)
Male	24 (35.8%)	27 (40.9%)	51 (38.3%)
Region			
North America	12 (17.9%)	9 (13.6%)	21 (15.8%)
Europe	45 (67.2%)	45 (68.2%)	90 (67.7%)
Asia	10 (14.9%)	11 (16.7%)	21 (15.8%)
Rest of the world	0	1 (1.5%)	1 (0.8%)
Race			
White	42 (72.4%)	45 (78.9%)	87 (75.7%)
Black or African American	3 (5.2%)	1 (1.8%)	4 (3.5%)
Asian	10 (17.2%)	11 (19.3%)	21 (18.3%)
Other^a^	3 (5.2%)	0	3 (2.6%)
Time since first appearance of HES symptoms (years), median (range)^b^	6.1 (0.5–39.5)	3.8 (0.5–36.7)	4.9 (0.5–39.5)
Time since HES diagnosis (years), median (range)^c^	1.9 (0.1–32.3)	2.0 (0.1–25.4)	1.9 (0.1–32.3)
Eosinophil count before OCS-responsiveness assessment and randomization: local result (cells µl^−1^), median (range)^d^	1,600 (1,000–10,250)	1,550 (1,000–25,150)	1,600 (1,000–25,150)
HES subtypes			
I-HES	49 (73.1%)	51 (77.3%)	100 (75.2%)
L-HES	5 (7.5%)	11 (16.7%)	16 (12.0%)
SO-HES^e^	10 (14.9%)	3 (4.5%)	13 (9.8%)
EGPA/HES overlap^f^	3 (4.5%)	0	3 (2.3%)
Unknown	0	1 (1.5%)	1 (0.8%)
HES organ involvement^g^			
Pulmonary	34 (50.7%)	42 (63.6%)	76 (57.1%)
Dermatologic	33 (49.3%)	37 (56.1%)	70 (52.6%)
Gastrointestinal	33 (49.3%)	30 (45.5%)	63 (47.4%)
Musculoskeletal	25 (37.3%)	24 (36.4%)	49 (36.8%)
Sinus	25 (37.3%)	20 (30.3%)	45 (33.8%)
Cardiac	7 (10.4%)	6 (9.1%)	13 (9.8%)
Neurological	6 (9.0%)	9 (13.6%)	15 (11.3%)
Other	11 (16.4%)	10 (15.2%)	21 (15.8%)
Number of organs involved^h^			
Median (range)	2 (1–5)	3 (1–5)	3 (1–5)
1	19 (28.4%)	17 (25.8%)	36 (27.1%)
2	15 (22.4%)	15 (22.7%)	30 (22.6%)
3	11 (16.4%)	11 (16.7%)	22 (16.5%)
4	15 (22.4%)	16 (24.2%)	31 (23.3%)
5	7 (10.4%)	7 (10.6%)	14 (10.5%)
Most bothersome HES symptom at baseline			
Fatigue	22 (32.8%)	30 (45.5%)	52 (39.1%)
Shortness of breath	20 (29.9%)	25 (37.9%)	45 (33.8%)
Cough	17 (25.4%)	19 (28.8%)	36 (27.1%)
General weakness	17 (25.4%)	12 (18.2%)	29 (21.8%)
Skin itching	13 (19.4%)	15 (22.7%)	28 (21.1%)
PROMIS Fatigue, median (range)^i^	55.1 (29.4–74.8)	56.4 (29.4–71.1)	55.1 (29.4–74.8)
Number of HES flares in the previous 12 months			
Median (range)	2 (0–12)	2 (1–12)	2 (0–12)
0	1 (1.5%)	0	1 (0.8%)
1	13 (19.4%)	13 (19.7%)	26 (19.5%)
2	28 (41.8%)	30 (45.5%)	58 (43.6%)
≥3	25 (37.3%)	23 (34.8%)	48 (36.1%)
Actively flaring at screening			
No	5 (7.5%)	7 (10.6%)	12 (9.0%)
Yes	62 (92.5%)	59 (89.4%)	121 (91.0%)
HES background therapy at baseline			
Systemic OCS^j^	47 (70.1%)	55 (83.3%)	102 (76.7%)
Median (range), mg day^−1^	5.0 (2.5–45.0)	6.8 (1.0–30.0)	5.0 (1.0–45.0)
≤10 mg day^−1^	43 (64.2%)	45 (68.2%)	88 (66.2%)
>10 to ≤20 mg day^−1^	4 (6.0%)	9 (13.6%)	13 (9.8%)
>20 mg day^−1^	0	1 (1.5%)	1 (0.8%)
Oral budesonide	3 (4.5%)	1 (1.5%)	4 (3.0%)
Cytotoxic//immunosuppressive therapies^k^	6 (9.0%)	5 (7.6%)	11 (8.3%)
Not on background systemic OCS^l^ or cytotoxic//immunosuppressive therapies^k^	12 (17.9%)	6 (9.1%)	18 (13.5%)
Other HES therapies^m^	45 (67.2%)	46 (69.7%)	91 (68.4%)

	Benralizumab (*n* = 67)	Placebo (*n* = 65)	Total (*N* = 132)
SF-36v2 physical component score, median (range)	46.1 (17.2–58.5)	43.6 (22.6–65.2)	–
SF-36v2 mental component score, median (range)	45.6 (17.8–64.5)	46.7 (15.8–61.5)	–
PGI-S category^n^			
No symptoms	4 (6.0%)	4 (6.2%)	8 (6.0%)
Very mild	10 (14.9%)	8 (12.3%)	18 (13.6%)
Mild	10 (14.9%)	11 (16.9%)	21 (15.9%)
Moderate	30 (44.8%)	19 (29.2%)	49 (37.1%)
Severe	10 (14.9%)	21 (32.3%)	31 (23.5%)
Very severe	3 (4.5%)	2 (3.1%)	5 (3.8%)

Data are *n* (%) unless otherwise indicated.

^a^Includes race categories ‘Native Hawaiian or other Pacific islander’, ‘American Indian or Alaska native’ and ‘Other’.

^b^The time to first appearance of HES symptoms (years) = (date of randomization − start date of HES worsening + 1)/365.25.

^c^Time since HES diagnosis (years) = (date of randomization − date of HES diagnosis + 1)/365.25.

^d^Eligibility was confirmed on the basis of local laboratory results.

^e^HES with involvement of a single organ system.

^f^HES with clinical features suggestive of EGPA (that is, asthma, chronic rhinosinusitis with nasal polyposis), but ANCA-negative and no history of documented or suspected vasculitis.

^g^Percentages do not equal 100%, patients may have had multiple organ involvement.

^h^One primary and up to four other organs could be selected by patients.

^i^Standardized *T*-score range for Short Form 7a is from 29.4 to 83.2; higher scores indicate greater fatigue severity.

^j^Prednisone equivalent.

^k^Cytotoxic//immunosuppressive therapies include, but are not limited to, hydroxyurea, cyclosporine, imatinib, methotrexate, tacrolimus and azathioprine.

^l^Systemic OCS includes oral budesonide. Four patients were taking oral budesonide; three in the benralizumab group and one in the placebo group.

^m^Other HES therapies include, but are not limited to, beclomethasone dipropionate, formoterol fumarate, omeprazole, salbutamol, tiotropium bromide, triamcinolone, acetonide and cetirizine.

^n^PGI-S is a single item to capture the patient’s perception of overall symptom severity.

ANCA, antineutrophil cytoplasmic antibodies; I-HES, idiopathic HES; L-HES, lymphocytic HES; SO-HES, single-organ HES.

### Primary endpoint

**Fig. 2:**
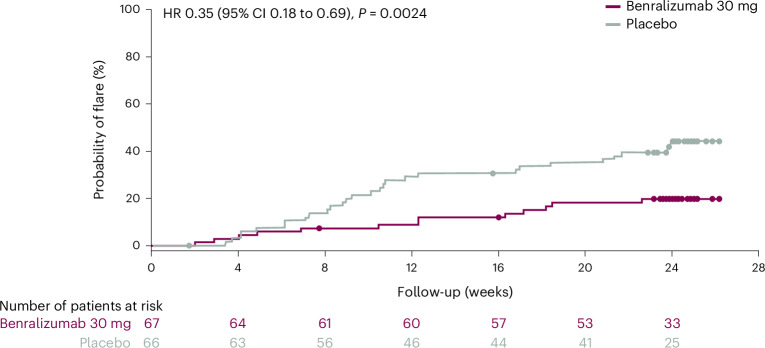
Time to first HES flare (primary endpoint). The time to first HES flare was analyzed using a stratified log-rank test (two-sided significance level of 0.05) adjusted for region. HR and 95% CIs were estimated using a Cox proportional hazards model with treatment group and region as covariates. For patients who did not experience a HES worsening/flare, the time to first HES worsening/flare was right censored at the end of the double-blind period corresponding to the earliest date of: the first benralizumab open-label dose, study day 183, the date of last contact and the data cutoff date. An HR <1 favors benralizumab. Flare was defined as HES clinical manifestations or laboratory abnormalities that resulted in an increase/burst of OCS ≥10 mg day^−1^ prednisolone equivalent for at least 2 days or an increase or addition of new cytotoxic and/or immunosuppressive therapy or hospitalization.

HES flare was observed in 13 patients (19.4%) receiving benralizumab and 28 patients (42.4%) receiving placebo. There was a delay in time to first HES flare in those receiving benralizumab versus placebo, with a statistically significant 65% reduction in risk (hazard ratio (HR) 0.35, 95% CI 0.18 to 0.69, *P* = 0.0024) (Fig. 2). Similar results were observed in a sensitivity analysis assessing whether changes in background therapy during the double-blind period impacted the time to first flare. Nine patients (five benralizumab, four placebo) with potentially relevant changes were censored at the point of medication change (Supplementary Fig. 2). Across predefined subgroups, the treatment effect of benralizumab versus placebo was consistent and favored benralizumab (Supplementary Fig. 3a).

### Key secondary endpoints

**Fig. 3:**
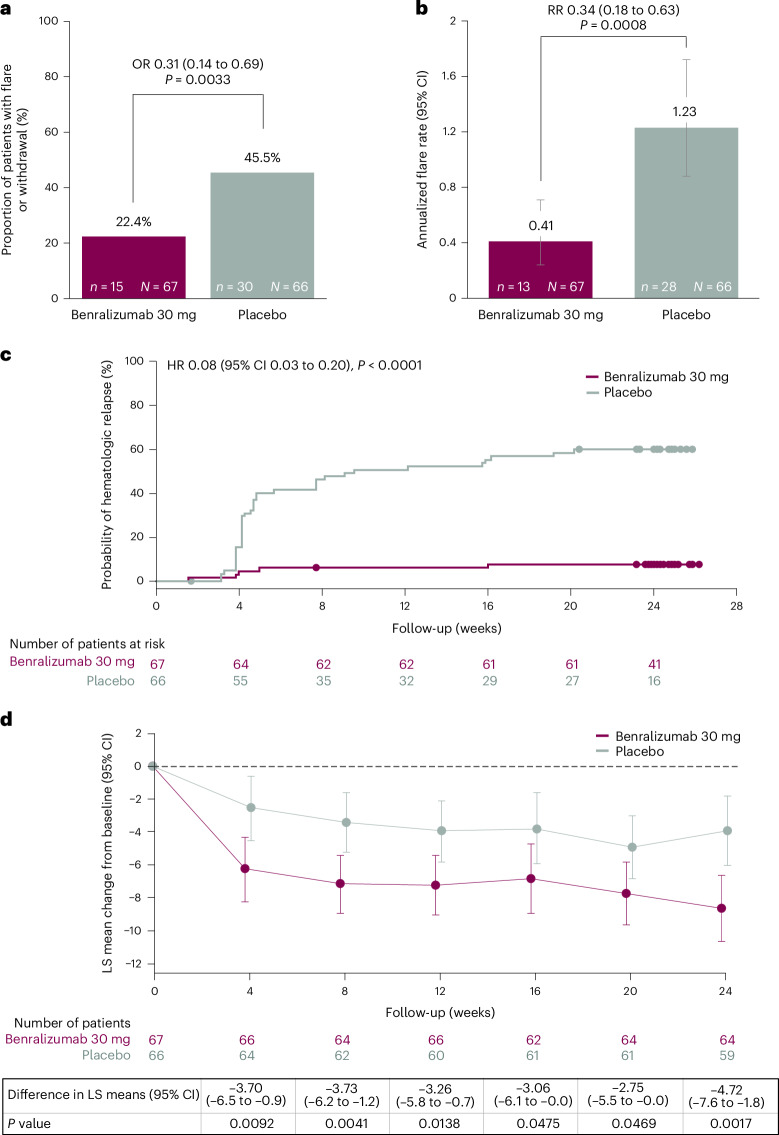
Key secondary endpoints. **a**, The proportion of patients with HES flares was analyzed using a logistic regression model with treatment group and region as covariates to estimate the OR and 95% CIs. This analysis included patients who withdrew from the study without having flares as an event; these patients were censored at the time of study withdrawal in the analysis of time to first flare. **b**, The number of HES flares (annualized rate) was analyzed using a negative binomial model with treatment group and region as covariates. The logarithm of follow-up time was used as an offset variable. Flare rates were estimated for each treatment group (error bars represent 95% CIs) and the RR and 95% CIs were calculated for benralizumab versus placebo. **c**, The time to first hematologic relapse (AEC ≥1,000 cells µl^−1^) was analyzed using a stratified log-rank test, adjusted for region. HR and 95% CIs were estimated using a Cox proportional hazards model with treatment group and region as covariates. Patients who did not experience hematologic relapse were right censored at the end of the double-blind period corresponding to the earliest date of: the first benralizumab open-label dose, study day 183, the date of last contact and the data cutoff date. **d**, The LS mean change from baseline to week 24 in PROMIS Fatigue scores was analyzed using a mixed model for repeated measures, with treatment group, baseline score, visit, region and treatment visit interaction as covariates. All available observations were included with no imputation for missing data, assuming missing data were missing at random. The standardized *T*-score range for Short Form 7a is from 29.4 to 83.2; higher scores indicate more severe fatigue. *P* values from week 4 to 20 are nominal. Hierarchical fixed sequence testing of key secondary endpoints was conducted in the order presented (**a**–**d**) at a two-sided significance level of 0.05. Error bars represent 95% CIs. Values of HR <1, OR <1, RR <1 and LS mean difference <0 favor benralizumab.

All endpoints met the prespecified hierarchical testing strategy (*P* < 0.05) and achieved statistical significance at the 1% level (Table 2). The proportion of patients who experienced a HES flare or withdrew from the study during the double-blind period was significantly lower in the benralizumab group compared to the placebo group: 22.4% (15/67) versus 45.5% (30/66), respectively (odds ratio (OR) 0.31, 95% CI 0.14 to 0.69, *P* = 0.0033) (Fig. 3a). This represented a 52% relative reduction in the proportion of patients experiencing a flare (rate ratio (RR) 0.48, 95% CI 0.29 to 0.80, *P* = 0.003). Significantly fewer HES flares occurred in those treated with benralizumab versus placebo (0.41 versus 1.23 flares per year, respectively), with a 66% reduction in the annualized rate of flares (RR 0.34, 95% CI 0.18 to 0.63, *P* = 0.0008) (Fig. 3b). A higher proportion of patients on benralizumab experienced no HES flare events during the 24-week double-blind period compared to placebo (80.6% (54/67) versus 57.6% (38/66), respectively). Fewer patients on benralizumab 13/67 (19.4%) experienced one flare, while 20/66 (30.3%) patients on placebo experienced one flare. No patients in the benralizumab group experienced more than one flare, while 8/66 (12.1%) in the placebo group had two flares.

**Table 2:** Summary of efficacy outcomes

Endpoint	Benralizumab (*n* = 67)	Placebo (*n* = 66)	Comparison (95% CI)	*P* value
Primary				
Time to first HES flare, *n* events (%)	13 (19.4%)	28 (42.4%)	HR 0.35 (0.18 to 0.69)^a^	0.0024
Key secondary				
Proportion of patients experiencing a flare or withdrawing by week 24, *n* (%)^b^	15 (22.4%)	30 (45.5%)	OR 0.31 (0.14 to 0.69)	0.0033
Annualized flare rate, flares per year (95% CI)^c^	0.41 (0.24 to 0.71)	1.23 (0.88 to 1.72)	RR 0.34 (0.18 to 0.63)	0.0008
Time to first hematologic relapse, *n* events (%)^d^	5 (7.5%)	39 (59.1%)	HR 0.08 (0.03 to 0.20)^a^	<0.0001
Change from baseline in PROMIS Fatigue score at week 24, LS mean (95% CI)^e^	−8.6 (–10.6 to –6.6)	−3.9 (–6.0 to –1.8)	LS means difference –4.72 (–7.64 to –1.80)	0.0017
Additional secondary				Nominal *P* value
Proportion of patients with hematologic relapse or withdrawing by week 24, *n* events (%)^b,d^	6 (9.0%)	42 (63.6%)	OR 0.05 (0.02 to 0.13)	<0.0001
Proportion of patients with AEC <500 cells µl^−1^ during the double-blind period, *n* (%)^b^	61 (91.0%)	8 (12.1%)	OR 87.9 (26.1 to 296.0)	<0.0001
Proportion of patients requiring an increase in systemic corticosteroids during the double-blind period, *n* (%)^b,f^	17 (25.4%)	32 (48.5%)	OR 0.35 (0.16 to 0.73)	0.005
Cumulative OCS dose over 24-week double-blind period (mg)	876.3	1221.2	LS means difference –344.9 (–637.6 to –52.3)	0.0213
	*n* = 64	*n* = 58		
Change from baseline in SF-36v2 PCS at week 24, LS mean (95% CI)^g^	6.7 (4.9 to 8.5)	2.2 (0.3 to 4.0)	LS means difference 4.54 (1.9 to 7.1)	0.0008
Change from baseline in SF-36v2 MCS at week 24, LS mean (95% CI)^g^	5.3 (3.2 to 7.3)	2.7 (0.5 to 4.8)	LS means difference 2.60 (–0.4 to 5.6)	0.0852

The double-blind period started from the date of randomization until the earliest date of: the first of benralizumab open-label dose, study day 183, the date of last contact or the data cutoff date. Values of HR <1, OR <1, RR <1 and LS mean difference <0 favor benralizumab.

^a^Estimated using a Cox proportional hazards model and analyzed with a stratified log-rank test.

^b^For proportion-based endpoints, the *P* value was derived from a Cochran–Mantel–Haenszel test, which constituted the primary analysis for the endpoint. The OR was estimated from a logistic regression model.

^c^A separate HES flare was considered if the start date of a flare was at least 14 days apart from the stop date of a preceding flare. Total follow-up time was defined as the time from randomization to the end of the double-blind period. Crude annual flare rate was calculated as 365.25 × (total number of flares/total follow-up time in days) within each treatment group. Annualized flare rates were estimated using a negative binomial model adjusted for region, with the logarithm of follow-up time included as an offset. Reported annualized flare rates represent model-estimated marginal rates.

^d^Hematologic relapse was defined when AEC post-baseline was ≥1,000 cells µl^−1^ for the first time.

^e^Standardized *T*-score range for Short Form 7a is from 29.4 to 83.2. A reduction in score indicates improvement.

^f^Included any increase of ≥1 mg prednisone-equivalent corticosteroid versus the previous day, where the reason for therapy was the disease under study *or* HES flare *or* for another condition with closely related symptoms.

^g^Estimated using a repeated-measures analysis of covariance. An increase in score indicates an improvement.

There was a significant delay in the time to first hematologic relapse: benralizumab patients were 92% less likely to relapse at any given point during the double-blind period versus placebo (HR 0.08, 95% CI 0.03 to 0.20, *P* < 0.0001). A clear separation between the treatment groups was demonstrated as early as week 4 (Fig. 3c). Fatigue was significantly improved in the benralizumab group versus the placebo group. The least squares (LS) mean difference in Patient-Reported Outcomes Measurement Information System (PROMIS) Fatigue scores between groups was –4.72 (95% CI –7.64 to –1.80, *P* = 0.0017) at week 24. The difference between groups in fatigue was already evident by the first timepoint measured (week 4) (Fig. 3d).

### Additional secondary endpoints

Following a protocol-specified OCS-responsiveness assessment, blood eosinophils were reduced at randomization in both placebo and benralizumab groups. After treatment, blood eosinophils reduced to near-complete depletion in the benralizumab group; however, in the placebo group, blood eosinophils increased and remained higher than the benralizumab group throughout the double-blind period (Supplementary Fig. 4).

Very few patients receiving benralizumab experienced hematologic relapse or withdrew from the study compared to those receiving placebo: 9.0% (6/67) versus 63.6% (42/66), respectively (OR 0.05, 95% CI 0.02 to 0.13, nominal *P* < 0.0001) (Supplementary Fig. 5).

Most patients on benralizumab sustained AEC <500 cells µl^−1^ for 24 weeks: 91.0% (61/67) compared to 12.1% (8/66) in the placebo group (OR 87.87, 95% CI 26.09 to 295.97, nominal *P* < 0.0001) (Supplementary Fig. 6).

Over the double-blind period, 25.4% (17/67) of patients receiving benralizumab versus 48.5% (32/66) receiving placebo required an increase in corticosteroid dose (OR 0.35, 95% CI 0.16 to 0.73, nominal *P* = 0.005) (Supplementary Fig. 7a). Fewer patients in the benralizumab versus placebo group required at least one corticosteroid increase during the double-blind period (Supplementary Fig. 7b). The LS mean cumulative OCS use during the 24-week double-blind period was lower in those on benralizumab than those on placebo (nominal *P* = 0.0213) (Supplementary Fig. 7c).

Improvements were observed in patients treated with benralizumab in both the physical and mental component summary scores of the Short Form-36 version 2 (SF-36v2) as early as week 12 (nominal *P* = 0.0104 and *P* = 0.0026, respectively) and were sustained to week 24 (nominal *P* = 0.0008 and *P* = 0.0852, respectively) (Supplementary Fig. 8).

Patient Global Impression of Severity (PGI-S) and Patient Global Impression of Change (PGI-C) data showed perceived improvements from baseline to week 24 among patients receiving benralizumab (Supplementary Figs. 9 and 10).

### PK and immunogenicity

Median benralizumab serum concentrations were generally similar between week 16 and 24 (Supplementary Fig. 11). Although the number of anti-drug antibody (ADA)-positive patients was too low to draw firm conclusions, no consistent impact of ADA on efficacy, safety or pharmacokinetics (PK) was observed (Supplementary Table 3).

### Safety

The proportion of patients experiencing any adverse event (AE) during the double-blind period was similar between treatment groups (benralizumab *n* = 43 (64.2%), placebo, *n* = 44 (66.7%)) (Table 3). The most common AEs were headache (11/67 (16.4%) benralizumab-treated patients and 5/66 (7.6%) placebo-treated patients), upper respiratory tract infection (5/67 (7.5%) and 5/66 (7.6%)) and coronavirus disease 2019 (4/67 (6.0%) and 4/66 (6.1%)). A full table of AEs is provided in Supplementary Table 4. Five (7.5%) benralizumab- and five (7.6%) placebo-treated patients experienced a serious AE, none of which were considered to be related to treatment. There was one death in the benralizumab group due to sepsis, which was not considered related to treatment by investigator assessment; please refer to the Supplementary Information for further details. Safety was consistent with the known safety profile of benralizumab.

**Table 3:** AEs during the double-blind treatment period

	Benralizumab (*n* = 67, Exp 30.6)	Placebo (*n* = 66, Exp 29.9)
Any AE	43 (64.2%)	44 (66.7%)
Any AE with outcome of death	1 (1.5%)	0
Any SAE (including events with outcome of death)^a^	5 (7.5%)	5 (7.6%)
Any AE leading to treatment discontinuation	1 (1.5%)	0
Most common AEs (≥5% in any arm), MedDRA preferred term		
Headache	11 (16.4%)	5 (7.6%)
Upper respiratory tract infection	5 (7.5%)	5 (7.6%)
Coronavirus disease 2019	4 (6.0%)	4 (6.1%)
Influenza-like illness	4 (6.0%)	0
Arthralgia	3 (4.5%)	5 (7.6%)
Nasopharyngitis	3 (4.5%)	4 (6.1%)
All SAEs^a^, MedDRA preferred term		
Hypereosinophilic syndrome	1 (1.5%)	1 (1.5%)
Chronic eosinophilic leukemia	1 (1.5%)	0
Dyspepsia	0	1 (1.5%)
Fetal death	0	1 (1.5%)
Gastrointestinal infection	0	1 (1.5%)
Hypersensitivity	1 (1.5%)	0
Incarcerated inguinal hernia	1 (1.5%)	0
Pneumonia, bacterial	0	1 (1.5%)
Sepsis	1 (1.5%)	0
Tubulointerstitial nephritis	0	1 (1.5%)

Data are presented as *n* (%).

^a^None of the SAEs was considered to be related to benralizumab. There was one death on benralizumab unrelated to treatment (sepsis). MedDRA version 28.0.

Exp, total on-study period (years) across all patients; MedDRA, Medical Dictionary for Regulatory Activities; SAE, serious AE.

### Post hoc analyses

As multiple organ involvement is present in many patients with HES, additional analyses were performed to assess the efficacy of benralizumab among patients with involvement of each major organ system, primary (Supplementary Fig. 3a) or other (Supplementary Fig. 3b). Benralizumab reduced the risk of the first flare versus placebo, regardless of the organ system involved.

## Discussion

In the phase 3 NATRON study, treatment with benralizumab delayed the time to first HES flare and resulted in a statistically significant 65% reduction in the risk of first HES flare compared to placebo. Fewer benralizumab-treated patients flared, and benralizumab therapy was also associated with a lower annualized flare rate and delayed time to first hematologic relapse compared to placebo. Benralizumab significantly improved fatigue, a key symptom of HES that negatively impacts health-related quality of life^24,25^, with improvements observed as early as 4 weeks and maintained to 24 weeks. Consistent with the known mechanism of action of benralizumab, as well as previous observations in small trials or cohorts of patients with HES^11,14,26^, durable near-complete depletion of blood eosinophils was observed in patients treated with benralizumab. Safety and tolerability results were consistent with the known safety profile for benralizumab, including in adolescents. Overall, the results of the NATRON study support the use of benralizumab as a therapy to manage patients with *FIP1L1::PDGFRA*-negative HES.

Strengths of the NATRON trial include the high retention rate as well as the inclusion of patients with varied HES disease subtypes and clinical manifestations (most commonly pulmonary, dermatologic and gastrointestinal involvement), which generally reflect disease presentation in the real-world patient population^27^. The results of the key secondary endpoints supported the primary observations. Importantly, the HES flare definition was related to clinical features without relying solely upon blood eosinophil counts, and included physical examination and assessment of symptoms, ensuring that the efficacy of benralizumab was not overestimated owing to its eosinophil-depleting mechanism of action, allowing for a more balanced comparison with placebo. The tapering of background therapy was not permitted during the double-blind period of the study, even when HES disease was well controlled. This enabled a clearer assessment of disease-related symptoms without confounding effects related to OCS withdrawal. The OCS-responsiveness assessment at screening served as a safety measure to ensure that flares could be managed clinically without the need for investigators to monitor eosinophil counts. Furthermore, it was evident that high-dose OCS bursts are not an effective therapeutic approach for HES, as eosinophil counts returned to elevated levels in the placebo group by week 4.

The NATRON efficacy results and trial population are similar to those observed in the phase 3 registrational trial for mepolizumab^9^. Although a standardized definition of severe HES is not yet available, the elevated eosinophil counts, frequency of flares in the prior 12 months and the number of organs involved at baseline collectively indicate that the characteristics of the patient population enrolled was suitable for evaluating the efficacy of a biologic in this trial. While there are no head-to-head studies in HES, switching to a different anti-IL-5/receptor biologic when a patient fails to respond to another can result in clinical improvement^11^; therefore, having both biologic options for HES will benefit some patients.

The OCS-sparing potential of benralizumab has been demonstrated in severe eosinophilic asthma and EGPA^21,22^. In the head-to-head phase 3 study comparing benralizumab and mepolizumab in EGPA, more patients receiving benralizumab during the double-blind period achieved complete discontinuation of OCS^22^. This observation was further supported by the results in the first year of the open-label extension (OLE), when the proportion of patients who discontinued OCS increased after switching from mepolizumab to benralizumab^28^.

The OCS-sparing ability of benralizumab has also been observed in phase 2 and small cohort studies in patients with HES^9,11,14,26^. Fewer benralizumab-treated patients in this study required increases in OCS doses; however, as patients were not permitted to taper their background OCS dose, no conclusions on the OCS-sparing ability of benralizumab can be drawn. The ongoing NATRON OLE will provide data on the long-term durability of efficacy of benralizumab in patients with HES, and will also provide insights on the ability of benralizumab treatment to reduce exposure to corticosteroids and cytotoxic/immunosuppressive therapies.

While the NATRON results provide important insights, there are some limitations to the study that should be acknowledged. First, the sample size was small owing to the rarity of the disease. Unfortunately, this limited the ability to draw conclusions on efficacy on the basis of subgroups, particularly in those with the L-HES subtype (in which no patients in the benralizumab group experienced a HES flare during the double-blind period). Although the NATRON trial population was broadly similar to that seen in the registrational mepolizumab trial^9^, the duration of HES since diagnosis (1.9 years) was shorter than that observed in mepolizumab trials (approximately 5.5 years). NATRON excluded patients with life-threatening HES and, therefore, the benefit of benralizumab in acute life-threatening situations requires further study. Given that the double-blind period of the study was limited to 24 weeks, long-term efficacy and safety outcomes will be assessed in the ongoing OLE.

In conclusion, these data reinforce the central role of eosinophils in the pathophysiology of HES and support targeting eosinophils as a therapeutic strategy. Benralizumab demonstrated efficacy and safety in the treatment of HES, with evidence of clinical benefit apparent at the earliest visits in the trial, thus offering perspectives of rapid disease control in patients with active disease manifestations. These results may therefore expand the potential therapeutic options for patients with this disease.

## Methods

### Study design

The NATRON study, a double-blind, 24-week, phase 3, randomized, placebo-controlled trial with an ongoing OLE, assessed the efficacy and safety of benralizumab in patients with HES (Supplementary Fig. 1). This study was conducted at 40 sites across 15 countries (Argentina, Austria, Belgium, China, Denmark, France, Germany, India, Israel, Japan, the Netherlands, Poland, South Korea, the UK and the USA).

All patients remained on stable background HES therapy during screening and the double-blind period; however, modifications were allowed if required for a HES flare (treatment intensification) or an AE (treatment de-escalation) thought to be due to background therapy. Stable background therapy included oral, topical, nasal or inhaled corticosteroids, immunosuppressive or cytotoxic agents, interferon-alpha (IFN-α), and other medications used to control HES and/or manage HES symptoms.

Patients who completed the 24-week double-blind period were eligible to enter an OLE where all patients received benralizumab. The end-of-study definition is described in the Supplementary Information.

The trial was conducted in accordance with the ethical principles of the Declaration of Helsinki and is consistent with the International Council for Harmonisation Good Clinical Practice guidelines, the applicable regulatory requirements and the AstraZeneca policy on bioethics. The protocol, protocol amendments and any other relevant documents were reviewed and approved by the Independent Ethics Committees/Institutional Review Boards listed in the Supplementary Information. All patients provided written informed consent. International Council for Harmonisation E6 Good Clinical Practice guidelines were followed. Patients enrolled in the study were not compensated for their participation; however, reasonable reimbursement of expenses incurred by the patients (for example, travel and parking) was provided if allowed by local regulations. This was stated clearly in the informed consent form.

An independent safety monitoring board, comprising two clinicians and a statistician, monitored overall patient safety. To inform study design and conduct, 60 patients with HES were surveyed online with support from the American Partnership for Eosinophilic Disorders patient advocacy group, Invitae and Covance Patient Engagement to provide insights on study participation, the placebo-controlled design, extended dosing, site visits and support strategies for long-term study engagement. The full protocol is publicly available at https://www.astrazenecaclinicaltrials.com/study/D3254C00001/. This trial is registered with ClinicalTrials.gov (NCT04191304) and the OLE is ongoing (closed to recruitment).

Patients were ≥12 years of age, had a diagnosis of HES (defined as history of persistent eosinophilia (>1,500 cells μl^−1^) without secondary cause on two examinations (≥1 month apart) and evidence of end-organ manifestations attributable to eosinophilia), were receiving documented stable HES therapy for ≥4 weeks before screening and were experiencing an HES flare at screening or had a history of ≥2 HES flares that required escalation in therapy within 12 months before screening.

Exclusion criteria were the presence of an *FIP1L1::PDGFRA* fusion tyrosine kinase gene rearrangement or other known imatinib-sensitive mutation; a confirmed diagnosis of EGPA or systemic mastocytosis; the presence of life-threatening HES complications, as judged by the investigator; a history of thrombotic complications, stroke or cardiac damage; a disease severity that made the patient inappropriate for inclusion; hypereosinophilia of unknown significance; a known, preexisting, clinically significant endocrine, autoimmune, metabolic, neurological, renal, gastrointestinal, hepatic, hematologic, respiratory or other systemic disorders not associated with HES that were uncontrolled with standard treatment and, in the opinion of the investigator, could have increased risk of patient safety, interfered with study outcomes or impaired completion of the study; a documented history of clinically significant cardiac damage; a known active liver disease at the time of study; a malignancy or history of malignancy within 5 years before screening; chronic or active infections requiring systemic treatment or clinically significant viral, bacterial or fungal infection within 4 weeks before visit 1; an untreated or inadequately treated helminth parasitic infection within 24 weeks before visit 1 without documented resolution; a known immunodeficiency disorder other than those attributable to OCS or HES-related therapy; a positive HIV test; any clinically significant abnormal findings on physical examination, vital signs, hematology or clinical chemistry during screening that, in the investigator’s opinion, could have posed a safety risk, influenced study results or impaired study completion; evidence of prior benralizumab treatment failure; treatment with injectable corticosteroids within 4 weeks before randomization; receipt of any investigational product within 30 days or five half-lives (whichever was longer) before visit 1 or concurrent participation in another interventional clinical study, excluding noninterventional registry or cohort studies; receipt of any marketed or investigational biologic within 4 months or five half-lives before informed consent, unless on stable background biologic therapy unlikely to interfere with safety or efficacy assessments; receipt of live attenuated vaccines within 30 days before visit 1; a history of hypersensitivity to any biologic therapy, corticosteroids or components of the investigational product; receipt of immunoglobulin or blood products within 30 days before visit 1; a known or suspected alcohol or substance abuse that could have interfered with protocol compliance; and pregnancy, breastfeeding or lactation at the time of the study. The full protocol can be accessed at https://www.astrazenecaclinicaltrials.com/study/D3254C00001/.

Following enrollment, eligible patients entered a 3-day screening period. To proceed to randomization, two criteria had to be met: first, an AEC ≥1,000 cells μl^−1^ at local laboratory testing on the date of enrollment, and second, a demonstration of corticosteroid responsiveness defined as an AEC <1,000 cells μl^−1^ after 2 days of OCS administration (1 mg kg^−1^ day^−1^ prednisone/prednisolone equivalent) given in addition to the patient’s background therapy for HES before randomization. This OCS-responsiveness assessment served as a safety measure to ensure that flares could be managed clinically without the need for investigators to monitor eosinophil counts, as eosinophil counts were blinded during the study. The OCS dose equivalency is shown in Supplementary Table 1.

Patients who met eligibility criteria at the end of the screening period were stratified by geographic region (North America, Europe, Asia and rest of the world) and HES flare status (active flare or historic flares at study entry). The inclusion of HES flare status as a stratification factor was introduced in June 2022; before this amendment, patients were required to be actively flaring at entry.

### Randomization and masking

All patients were centrally assigned to a randomized study treatment using Interactive Web Response Systems (IWRS)/Interactive Voice Response Systems (IVRS). As patients became eligible for randomization, unique randomization codes were assigned sequentially in each stratum from a randomization list prepared by a computerized system provided on behalf of AstraZeneca. The randomization sequence was computer-generated centrally using a permuted block design with a fixed block size of 4 and stratified by geographic region (North America, Europe, Asia and rest of the world) and HES flare status at screening. Patients, sponsor, site staff and investigators were blinded to treatment allocation and to patients’ blood and biopsy leukocyte counts during the double-blind treatment period and up to week 4 of the OLE. All packaging and labeling ensured blinding for all sponsor and investigational site staff. The following personnel had access to the randomization list during the study: those generating the randomization list, personnel at the IWRS/IVRS company, AstraZeneca’s supply chain department, drug safety services representatives (data entry site case handlers), the bioanalytical laboratory performing the PK sample analysis, the independent statistical data analysis center, the unblinded programmer and the unblinded medical monitor. The IWRS/IVRS provided the investigators with the kit identification number to be allocated to the patient at the dispensing visit.

### Procedures

Patients were randomly assigned 1:1 into the 24-week double-blind period where they received benralizumab 30 mg (accessorized prefilled syringe) subcutaneously every 4 weeks or a matching placebo, in addition to the patient’s background therapy for HES. The rationale for benralizumab dosing is described in the Supplementary Information.

In addition to screening and scheduled study visits every 4 weeks, patients were advised to contact the study site each time they thought their symptoms were worsening and attend the site for a flare visit assessment. If study treatment was discontinued early, patients attended a discontinuation visit 4 ± 1 weeks after the last dose.

HES flares were assessed by the investigator at all scheduled or flare visits. Vital signs and blood samples were collected at screening and all scheduled and flare site visits; for scheduled dosing visits, samples were taken before the administration of study treatment.

Patient-reported outcome assessments were completed by patients using an electronic device at study site visits before other study procedures. Patient-reported outcome assessments included: (1) PROMIS Fatigue short form 7a, measured at screening and scheduled study visits (that is, every 4 weeks); (2) SF-36v2 (acute recall), measured at screening, visit 6 (week 12) and visit 9 (week 24); (3) PGI-S, measured at screening and scheduled study visits (every 4 weeks); and (4) PGI-C, measured at visit 4 (week 4) and scheduled study visits (every 4 weeks).

PK and immunogenicity assessments were conducted at screening and pre-dose at scheduled visits 4 (week 4), 5 (week 8), 7 (week 16) and 9 (week 24). AEs included events reported between screening (visit 1) and last contact with the patient. Serious AEs included events recorded from written informed consent throughout the duration of the study.

### Outcomes

The primary endpoint was time to first HES flare during the 24-week, double-blind treatment period. A flare was defined as HES clinical manifestation or laboratory abnormality resulting in an increase of OCS ≥10 mg day^−1^ prednisone equivalent for ≥2 days, or an increase or addition of a new cytotoxic and/or immunosuppressive therapy, or hospitalization. Flares were assessed by the investigator through complete or brief physical examinations, an investigator-led HES symptom interview, laboratory assessments and other routine safety assessments; please refer to the Supplementary Information for further details. If patients were unable to attend the study site for flare assessment, medical records were collected and an investigator-led HES symptoms interview was recommended. Time to first HES flare was calculated as the number of days from the date of randomization to the start date of the first flare event, plus 1 day. The start date of HES flare was defined as the first day of increased dose/burst of OCS, first day of any increase or addition of new cytotoxic and/or immunosuppressive therapy, or date of hospital admission, whichever occurred first.

Secondary endpoints that were multiplicity protected within the prespecified statistical testing hierarchy were defined as ‘key’. Key secondary endpoints were: (1) the proportion of patients with HES flares, with those who withdrew from the study without having experienced a flare considered as having had a flare event; (2) the annualized rate of HES flares, assessed over a maximum follow-up period of 24 weeks or, for patients lost to follow-up, the follow-up time was defined as the duration from randomization to the last timepoint at which flare status could be evaluated, with distinct flares defined as those with onset occurring ≥14 days after the resolution of the previous flare; (3) the time to first hematologic relapse (AEC ≥1,000 cells μl^−1^), calculated as the number of days from the date of randomization to the start date of first hematologic relapse plus 1 day; and (4) the change from baseline to week 24 in PROMIS Fatigue, with a standardized total score calculated for each visit over the double-blind period.

Other secondary endpoints included the proportion of patients with hematologic relapse (including those who withdrew from the study) during the double-blind period, the proportion of patients with AEC <500 cells µl^−1^ for 24 weeks, the proportion of patients requiring an increase in corticosteroid dose at any time during the double-blind period and other patient-reported outcomes (SF-36v2, PGI-S and PGI-C).

PK was assessed through benralizumab serum concentrations and immunogenicity assessed through ADA assays and neutralizing antibody testing, as previously described^29^. Safety was assessed through reporting of AEs, serious AEs, vital signs and clinical laboratory variables.

### Statistical analysis

All efficacy endpoints, demographics and baseline characteristics were analyzed using the full analysis set, which included all randomized patients who received ≥1 dose of study treatment according to the intention-to-treat principle, irrespective of adherence to the protocol and continued trial participation.

The primary analysis data cutoff was to occur after 38 patients had a first HES flare event and all randomized patients had completed the 24-week double-blind period. It was estimated that approximately 38 first HES flare events during the double-blind period were required to detect a statistically significant difference between treatment groups at the two-sided 5% significance level with approximately 80% power if the true treatment effect is an HR of 0.389 (equivalent to 30% of patients receiving benralizumab experiencing an event by the end of the double-blind period versus 60% of patients receiving placebo). On the basis of these assumptions, a sample size of approximately 120 was expected, although recruitment could continue beyond this to provide confidence that sufficient events would be observed once complete follow-up was achieved. A sensitivity analysis was conducted to assess the impact of patients changing systemic background therapy before HES flares, by censoring any patients with systemic OCS or immunosuppressive therapy changes that were considered to have a potential to impact the chance of the patient flaring. Please refer to the Supplementary Information for further details on the sensitivity analysis. Subgroups were analyzed for the primary endpoint, and included sex, age, region (a stratification factor), race, HES subtype (based on investigator assignment), baseline blood eosinophil counts, baseline OCS dose, HES flare status (a stratification factor), primary organ involvement and time since HES diagnosis.

To account for multiplicity, if the primary endpoint was found to be statistically significant, key secondary endpoints were tested using a hierarchical fixed sequence approach at two-sided 0.05 in the following order: (1) the proportion of patients who experience a HES flare during the double-blind period; (2) the number of HES flares (annualized rate) during the double-blind period; (3) the time to first hematologic relapse during the double-blind period; and (4) the change from baseline in PROMIS Fatigue score at week 24.

Time-to-event endpoints were analyzed using a stratified log-rank test, adjusted for region. HRs and 95% CIs were estimated using a Cox proportional hazards model with treatment group and region as covariates. For patients who had not experienced the event, the time to event was censored at the end of the double-blind period corresponding to the date of the first of benralizumab open-label dose, study day 183, the date of last contact or the data cutoff date, whichever occurred first.

The proportion of patients with a HES flare was analyzed using a logistic regression model with treatment group and region as covariates to obtain the OR and 95% CIs (withdrawals were included as flare events in the primary analysis). The annualized rate of flares during the double-blind period was analyzed using a negative binomial model. The logarithm of the follow-up time was used as an offset variable in the model. The model included covariates of treatment group and region to obtain estimates of the flare rates in each treatment group and the rate ratio for benralizumab versus placebo.

For continuous change from baseline endpoints, including changes in PROMIS Fatigue standardized *T*-scores, data were analyzed using a mixed model for repeated measures, with treatment group, baseline score, visit, region and treatment visit interaction as covariates. No imputations were performed for missing data; all available observations were included in the analyses, which assumed missing data were missing at random. Safety was analyzed descriptively on the basis of the safety analysis set, which included all patients who received ≥1 dose of study treatment. The PK analysis set included all patients who received ≥1 dose of benralizumab with ≥1 quantifiable serum PK observation post-first dose. All data analyses were performed with SAS System (SAS Institute Inc.) software. Please refer to the Supplementary Information for additional details on statistical analyses.

### Reporting summary

Further information on research design is available in the Nature Portfolio Reporting Summary linked to this article.

## Online content

Any methods, additional references, Nature Portfolio reporting summaries, source data, extended data, supplementary information, acknowledgements, peer review information; details of author contributions and competing interests; and statements of data and code availability are available at 10.1038/s41591-026-04315-8.

## Supplementary information

Supplementary Information Members of the NATRON Study Group, Supplementary Methods, narrative of fatality in the study, Supplementary Figs. 1–11 and Supplementary Tables 1–4.

Reporting Summary

## Data Availability

Data underlying the findings described in this Article can be requested in accordance with AstraZeneca’s data sharing policy available via AstraZeneca at https://astrazenecagrouptrials.pharmacm.com/ST/Submission/Disclosure. Data for studies directly listed on Vivli are available via Vivli at https://www.vivli.org. Data for studies not listed on Vivli are available via Vivli at https://vivli.org/members/enquiries-about-studies-not-listed-on-the-vivli-platform/. The AstraZeneca Vivli member page outlining further details is also avialable via Vivli at https://vivli.org/ourmember/astrazeneca/.
