# Author Correction: Benralizumab versus placebo for hypereosinophilic syndrome: a randomized, placebo-controlled phase 3 trial

**DOI:** 10.1038/s41591-026-04425-3

**Published:** 2026-05-05

**Authors:** Princess U. Ogbogu, Florence Roufosse, Praveen Akuthota, Piotr Kuna, Matthieu Groh, Andreas Reiter, Akira Yokota, Salman H. Siddiqui, Pim G. N. J. Mutsaers, Bing Li, Paneez Khoury, Lila M. Bahadori, Artur Bednarczyk, Gerben Bouma, Laura G. Brooks, Jorge Ferreira, Hanna Grindebacke, Calvin N. Ho, Priya Jain, Rebecca L. Palmer, Maria L. Jison, Amy D. Klion

**Affiliations:** 1 Division of Pediatric Allergy, Immunology, and Rheumatology, Department of Pediatrics, University Hospitals Rainbow Babies and Children’s Hospital, Case Western Reserve University School of Medicine, Cleveland, OH USA; 2 Department of Internal Medicine, Hôpital Universitaire de Bruxelles – Site Erasme, Université Libre de Bruxelles, Brussels, Belgium; 3 Division of Pulmonary, Critical Care, Sleep Medicine and Physiology, Department of Medicine, University of California San Diego, La Jolla, CA USA; 4 Division of Internal Medicine Asthma and Allergy, Medical University of Lodz, Lodz, Poland; 5 Universtié de Versailles Saint-Quentin-en-Yvelines, Montigny-Le-Bretonneux, France; National Referral Center for Hypereosinophilic Syndromes, Department of Internal Medicine, Clinical Immunology and Hematology, Hôpital Foch, Suresnes, France; 6 Department of Hematology and Oncology, University Hospital Mannheim, Heidelberg University, Mannheim, Germany; 7 Department of Hematology, Chiba Aoba Municipal Hospital, Chiba, Japan; 8 NIHR Imperial Biomedical Research Centre, National Heart and Lung Institute, Royal Brompton and Hammersmith Hospitals, Imperial College London, London, UK; 9 Department of Hematology, Erasmus University Medical Center, Rotterdam, the Netherlands; 10 State Key Laboratory of Experimental Hematology, National Clinical Research Center for Blood Diseases, Haihe Laboratory of Cell Ecosystem, Institute of Hematology and Blood Diseases Hospital, Chinese Academy of Medical Sciences and Peking Union Medical College, Tianjin, China; 11 MDS and MPN Center, Institute of Hematology and Blood Diseases Hospital, Chinese Academy of Medical Sciences and Peking Union Medical College, Tianjin, China; 12 Laboratory of Parasitic Diseases, National Institute of Allergy and Infectious Diseases, National Institutes of Health, Bethesda, MD USA; 13 Respiratory and Immunology, BioPharmaceuticals R&D, AstraZeneca, Gaithersburg, MD USA; 14 Respiratory and Immunology, BioPharmaceuticals R&D, AstraZeneca, Warsaw, Poland; 15 Translational Science and Experimental Medicine, Respiratory and Immunology, BioPharmaceuticals R&D, AstraZeneca, Cambridge, UK; 16 Respiratory and Immunology, BioPharmaceuticals R&D, AstraZeneca, Cambridge, UK; 17 Respiratory and Immunology, BioPharmaceuticals R&D, AstraZeneca, Gothenburg, Sweden; 18 Respiratory and Immunology, BioPharmaceuticals Medical, AstraZeneca, Cambridge, UK

**Keywords:** Immunology, Medical research

Correction to: *Nature Medicine* 10.1038/s41591-026-04315-8, published online 31 March 2026.

In the version of this article initially published, there was an error in Table 1 where the PROMIS Fatigue median for the total population, now reading “55.1” appeared originally as “51.1.” In addition, errors in Supplementary Table 2 values have now been updated as follows: Eosinophil count median + Benralizumab (n = *67*) has been changed from 1,070.0 (230–5,620) to 1,085.0 (360–5,620); Eosinophil count median + Total (*N* = 133) has been changed from 1,120.0 (60–20,910) to 1,100.0 (60–20,910); and Prior HES-related biologic therapy in the past 12 months for Omalizumab + Benralizumab (*n* = 67) has been changed from 2 (1.5%) to 2 (3.0%). The tables have been updated in the HTML and PDF versions of the article.

