# Supplementary material for: Benralizumab versus placebo for hypereosinophilic syndrome: a randomized, placebo-controlled phase 3 trial
Source: Nat Med. 2026 Mar 31;32(6):2017–25. doi: 10.1038/s41591-026-04315-8 (PMC13278967; doi:10.1038/s41591-026-04315-8)
Supplement: Supplementary file 1 — Members of the NATRON Study Group, Supplementary Methods, narrative of fatality in the study, Supplementary Figs. 1–11 and Supplementary Tables 1–4. [file 41591_2026_4315_MOESM1_ESM.pdf]

nature medicine

Supplementary information https://doi.org/10.1038/s41591-026-04315-8

Benralizumab versus placebo for hypereosinophilic syndrome: a randomized, placebo-controlled phase 3 trial

In the format provided by the authors and unedited

Supplementary Appendix

Members of the NATRON Study Group 2

Independent Ethics Committees/Institutional Review Boards consulted 13

Supplementary methods 15

Rationale for benralizumab dose 15

End-of-study definition 15

HES flare assessment 15

Sensitivity analysis of the primary endpoint 16

Statistical methods: Additional secondary endpoints and post hoc analyses 16

Supplementary safety results 19

Narrative of fatality reported in this study 19

Supplementary figures 20

Supplementary Fig. 1 | NATRON study design 20

Supplementary Fig. 2 | Time to first HES flare (sensitivity analysis: patients changing systemic background therapy before HES flare) 21

Supplementary Fig. 3 | Subgroup and post hoc analyses of time to first HES flare 22

Supplementary Fig. 4 | Blood eosinophil counts over the double-blind period 24

Supplementary Fig. 5 | Proportion of patients with hematologic relapse during the double-blind period 25

Supplementary Fig. 6 | Proportion of patients with sustained AEC <500 cells/μL for 24 weeks 26

Supplementary Fig. 7 | Systemic corticosteroid use during the double-blind period* 27

Supplementary Fig. 8 | LS mean change from baseline in SF-36v2 component scores 29

Supplementary Fig. 9 | PGI-S category from baseline through to Week 24 30

Supplementary Fig. 10 | PGI-C category from Week 4 through to Week 24 31

Supplementary Fig. 11 | Benralizumab serum concentrations (PK analysis set) 32

Supplementary tables 33

Supplementary Table 1 | Systemic corticosteroid dose equivalences 33

Supplementary Table 2 | Additional baseline characteristics 34

Supplementary Table 3 | Anti-drug antibody response to benralizumab 36

Supplementary Table 4 | Adverse events by preferred term 37

Members of the NATRON Study Group

Name	Centre	Country
**Gabriel Gattolin**<br>Angelina Fracaroli	Centro Respiratorio Infantil	Argentina
**Judith M. Loeffler-Ragg**<br>Birgit Sailer<br>Thomas Sonnweber	Med. Universität Innsbruck, Universitätsklinik für Innere VI	Austria
**Vito Sabato**<br>Michiel Beyens<br>Didier Ebo<br>Alessandro Toscano	Antwerp University Hospital Belgium, University of Antwerp<br>Universitair Ziekenhuis Antwerpen	Belgium
**Florence Roufosse**<br>Caroline Carpentier	Clinique Universitaire Bruxelles Hôpital Erasme, Université Libre de Bruxelles	
**Hu Zhou**<br>Mengjuan Li<br>Xuewen Song	The Affiliated Cancer Hospital of Zhengzhou University & Henan Cancer Hospital<br>Henan Cancer Hospital	China
**Bing Li**<br>Zhijian Xiao<br>Lijuan Pan<br>Zefeng Xu	State Key Laboratory of Experimental Hematology, National Clinical Research Center for Blood Diseases, Haihe Laboratory of Cell Ecosystem, Institute of Hematology & Blood Diseases Hospital, Chinese Academy of Medical Sciences & Peking Union Medical College, Tianjin 300020, China.<br>MDS and MPN Centre, Institute of Hematology and Blood Diseases Hospital, Chinese Academy of Medical Sciences & Peking Union Medical College, Tianjin, China<br>Institute of Hematology and Blood Diseases Hospital, Chinese Academy of Medical Sciences	
**Kai Shen**<br>Chenlu Yang	West China Hospital, Sichuan University	
**Zheng Wei**<br>Yating Jiang<br>Xiaona Wang<br>Yangyang Wang<br>Yuxin Zhang	Zhongshan Hospital, Fudan University	
**Christen Lykkegaard Andersen**<br>Daniel El Fassi	Department of Hematology, Copenhagen University Hospital, Rigshospitalet, Denmark & Centre for	Denmark

Kirsten Grønbæk	General Practice, Department of Public Health, University of Copenhagen, Denmark	
**Guillaume Lefèvre**<br>Emmanuel Ledoult<br>Giorgia Venturelli	Hôpital Claude Huriez	France
**Jean-Emmanuel Kahn**<br>Felix Ackermann<br>Priscille Couture<br>Matthieu Groh<br>Benjamin Hugues<br>Romain Paule<br>Julien Rohmer<br>Mathilde Roumier	Hôpital Foch	
**Stanislas Faguer**<br>Julie Belliere<br>Alienor Galinier	University Hospital of Toulouse	
**Thierry Martin**<br>Aurelien Guffroy<br>Anne-Sophie Korganow<br>Vincent Poindron	Hôpitaux Universitaires de Strasbourg - Hôpital Civil	
**Jean-Francois Viallard**<br>Felix Blaison<br>Irene Machelart<br>Henry Dupuy<br>Pauline Durand<br>Carine Greib<br>Estibaliz Lazaro<br>Cedric Leonard<br>Camille Prot-Leurent<br>Etienne Rivière<br>Manon Roucoules<br>Claire Tinevez	Hôpital du Haut Lévêque	
**Andreas Reiter**<br>Karin Bonatz	Medizinische Fakultät Mannheim der Universität Heidelberg	Germany

Sebastian Kreil<br>Juliana Schwaab		
**Bastian Walz**<br>Bernhard Hellmich<br>Anke Reichelt de Tenorio<br>Ulrike Schlenker	Medius Clinics	
**Rohit Kumar**<br>Ganesh Narwade	Vardhman Mahavir Medical College & Safdarjung Hospital	India
**Piyush Arora**<br>Sanjiv Maheshwari<br>Jai Prakash Narayan	Jawahar Lal Nehru Medical College	
**Deepali J Kamdar**	Jaydeep Hospital	
**David Hagin**<br>Shira Benor<br>Shmuel Kivity<br>Dikla Adir Levanon	Tel Aviv Sourasky Medical Center	Israel
**Yossi Rosman**<br>Anat Cohen<br>Ronit Confino-Cohen	Meir Medical Centre	
**Miguel Stein**<br>Mary Isarelson<br>Adi Ovadia<br>Ester Rabin	Edith Wolfson Medical Centre, Tel Aviv University	
**Elena Mishchenko**<br>Mouna Ballan Haj<br>Iveta Mintsman<br>Shoshan Perek<br>Meir Preis<br>Louise Evelyne Shabad<br>Olga Valkovsky<br>Amir Warwar<br>Ibrahim Zoubi	Carmel Medical Centre	
**Daniel Elbirt**<br>Ilan Asher<br>Keren Mahlab-Guri	Kaplan Medical Center	

Shay Nemet<br>Shira Rosenberg Bezalel		
**Cristina Papayannidis**<br>Jacopo Nanni<br>Chiara Sartor<br>Antonio Curti<br>Gianluca Cristiano<br>Stefania Paolini	IRCCS Azienda Ospedaliero-Universitaria di Bologna - Istituto di Ematologia “Seràgnoli”, Bologna	Italy
**Naoto Azuma**<br>Kiyoshi Matsui<br>Masao Tamura<br>Takeo Abe<br>Kota Azuma<br>Tetsuya Furukawa<br>Teppei Hashimoto<br>Yuko Minagawa<br>Mai Morimoto<br>Mai Nakano<br>Kazuteru Noguchi<br>Chie Ogita<br>Yuko Ohno<br>Suzu Ueda<br>Takahiro Yoshikawa	Hyogo College of Medicine Hospital	Japan
**Akira Yokota**<br>Seito Iwai<br>Yuki Kasuya<br>Yoshihisa Kobayashi<br>Yuhei Nagao<br>Mayuko Negishi<br>Masahiro Onoda<br>Katsuhiro Shono	Chiba Aoba Municipal Hospital	
**Noriyoshi Ogawa**<br>Kumiko Shimoyama	Hamamatsu University School of Medicine	
**Nobuhiro Ohno**<br>Seiichiro Kobayashi	JOHAS, Kanto Rosai Hospital	

**Toshikazu Kano**<br>Yoshinori Masui<br>Naonori Tsuda	National Center for Global Health and Medicine - Kohnodai Hospital	
**Toshiyuki Kitano**<br>Akiko Aiba<br>Kiwamu Doi<br>Shojiro Inano<br>Naoto Kawasaki<br>Seigi Oshima<br>Yoshio Okamoto<br>Sho Shibata<br>Mitsuhiro Shimada<br>Sumie Tabata<br>Kohei Takeda<br>Yoko Takiuchi<br>Kazuyo Yamamoto	The TazukeKofukai Medical Research Institute, Kitano Hospital	
**Hiroshi Fujii**<br>Tomonori Ishii<br>Yusho Ishii<br>Yosuke Hoshi<br>Tomoaki Machiyama<br>Hiroko Sato<br>Tsuyoshi Shirai	Tohoku University Hospital	
**Pim GNJ Mutsaers**<br>Anna Aalbers<br>Jeanette K. Doorduijn<br>Mandy N. Lauw<br>Minke A. E. Rab<br>Yasmina Serroukh<br>Nicolette van der Sande<br>Ruben van Dijck	Erasmus Medisch Centrum	Netherlands
**Piotr B. Kuna**<br>Magdalena Adamczewska<br>Piotr Damianski<br>Katarzyna Jarmakowska	Klinika Chorob Wewnetrznych, Astmy i Alergii, Uniwersytet Medyczny Lodz, Poland<br>SP ZOZ Uniwersytecki Szpital Kliniczny nr 1	Poland

Julia Wnuk		
**Marta Chelminska**<br><br>Marika Gawinowska<br><br>Lucyna Górska<br><br>Ewelina Katarzyna Harceko-Zielinska<br><br>Jan Romantowski<br><br>Beata Wajda<br><br>Katarzyna Świętnicka	Uniwersyteckie Centrum Kliniczne w Gdańsku	
**Piotr Łacwik**<br><br>Jolanta Kozlowska-Murawska<br><br>Anna Moscicka<br><br>Dominika Ochab Krupnik	Wojewódzki Szpital Specjalistyczny im. Św. Rafała w Czerwonej Górze	
**Tae-Bum Kim**<br><br>You Sook Cho<br><br>Yeonhee Kim<br><br>Hyouk-Soo Kwon<br><br>Ji-Hyang Lee<br><br>Ji Yoon Oh<br><br>Woo-Jung Song	Asan Medical Center	South Korea
**Salman Siddiqui**<br><br>Lydia Finney<br><br>Beverly Kowlessar<br><br>Harold Wilson-Morkeh	Imperial College Healthcare NHS Trust	United Kingdom
**Anna Kovalszki**<br><br>Cem Akin<br><br>Sara S. Ellingwood	University of Michigan Allergy and Immunology Division<br><br>Allergy Specialty Clinic and Food Allergy Clinic at Domino's Farms	United States
**Peter B. Bressler**<br><br>Anne Collier<br><br>Katherine Elizabeth Prince<br><br>Jessica Shier	Duke Allergy and Pulmonary South Durham	
**Casey Curtis**<br><br>Chris Brooks	Ohio State University Wexner Medical Center	
**Princess Ogbogu**<br><br>Kathryn Ruda Wessell	Division of Pediatric Allergy, Immunology, and Rheumatology, Department of Pediatrics, University Hospitals Rainbow Babies and Children's Hospital, Cleveland, Ohio	

	Case Western Reserve University School of Medicine, Cleveland, Ohio	
**Praveen Akuthota**<br>Damaris Diaz	Division of Pulmonary, Critical Care, Sleep Medicine & Physiology, Department of Medicine, University of California San Diego, La Jolla, CA, USA	
**Mili Shum**<br>Gerald J. Gleich<br>Sean Wentland	University of Utah	
**Frances Eun-Hyung Lee**	Emory University Hospital	
**Amy D. Klion**<br>Charles Anderson<br>Thomas Brown<br>Gregory M. Constantine<br>Paneez Khoury<br>Celeste G. Nelson	National Institute of Allergy and Infectious Diseases	
Leanne Amery<br>Patricia Awori<br>Eileen Babcock<br>Lila M. Bahadori<br>Harry Bansal<br>Peter Barker<br>Nicole Barnor<br>Prashanth Basavanna<br>Saran Baskaran<br>Artur Bednarczyk<br>Kimberly Belknap<br>Assia Bensalem<br>Magnus Bergman Svärd<br>Gaweł Bojanowski<br>Gerben Bouma<br>Joanna Branicka<br>Laura G. Brooks<br>Ron Chen<br>Monica Chiaramonte	AstraZeneca (current or former employees)	

Robert Corbé<br>Natalia Dąbrowska<br>Gina D'Angelo<br>Catherine Datto<br>Carlo dela Seña<br>Beth Duncan<br>Kasia Dobaj<br>James Eaton<br>Ulrika Emerath<br>Katiana Fenelon<br>Jorge Ferreira<br>Martin Fredholm<br>Agnieszka Górnicka<br>Jacek Gregorczyk<br>Dianne Griffis<br>Hanna Grindebacke<br>Maria Härdelin<br>Shahram Hedjazifar<br>Rachel Hewitt<br>Calvin N. Ho<br>Shan Huang<br>Shabeena Huda<br>Agata Izydorczyk<br>Priya Jain<br>Maria L. Jison<br>Jenny Jonasson<br>Rohit Katial<br>Paulina Kazimierak<br>Matthew Keith<br>Vitalii Khomenko<br>Ewelina Kister<br>Anna Klatkowska<br>Kamil Kmita<br>Zuzanna Krakowska<br>Tatyana Kremenets	

Bartosz Kubiak<br>Anita Kukiełka<br>Yulia Kurdyukova<br>Aadarsh Lal<br>Katie Lee<br>Andrew Lee<br>Caron Lloyd<br>Pradeep Lukka<br>Rui Ma<br>Dorota Makara<br>Natalya Makulova<br>Writwik Mandal<br>Mary Kay Margolis<br>Artur Mark-Pawłowicz<br>Richard Martin<br>Melissa Marvel<br>Gaëll Mayer<br>Christopher McCrae<br>Margaret Melville<br>Ashwitha Naik<br>Laura Needleman<br>Michał Niziołek<br>Brad Nohe<br>Sean O'Quinn<br>Alejandra Ocampo<br>Rebecca L. Palmer<br>Jing Peattie<br>Urszula Piątkowska<br>Ioannis Psallidas<br>Abid Raza<br>Yasa Reddy<br>Ana Roman<br>Tim Ruckh<br>Rocío Salazar<br>Julia Samson	

Magdalena Sendek<br>Vivian H Shih<br>Jaskaran Singh Gill<br>Dariusz Słoboda<br>Anna Sokolnicka<br>Aleksandra Świączkowska<br>Katarzyna Szewczyk<br>Karol Szmyt<br>Hajime Takahashi<br>Anita Tałanda<br>Karolina Tarczyńska<br>Bartłomiej Twardowski<br>Anjali Vidhyadharan<br>Claire Walton<br>Patrycja Wąsikiewicz<br>Nicholas White<br>Wendy I White<br>Darlene Wilson<br>Oliwia Zachara<br>Monika Zapaśnik<br>Sofia Zetterstrand<br>Emilia Zubrzycka-Świątek	
Shazhad Aslam<br>Dwaine Banton<br>Marie Barbelivien<br>Priyanka Bhosale<br>Andrea Buschiazzo<br>Remie De Crock<br>Nathalie DeJaegher<br>Angela Farrar<br>Harmoni Gilmore<br>Marie-Christelle Goodyear<br>Ute Huebner-Otun<br>Lucas Laserna<br>Anusha Mallavarapu	Fortrea

Mickael Mouret<br>Madhura Pai<br>Alessandra Romei<br>Ian Saliendra<br>Abhijit Shinde<br>Monica Shutz<br>Anja Tonnes Madsen<br>Ada Xu<br>Mena Yacoub	
Andrea Hernández<br>Olivier Laban	Clario
Janine Micheli	Thermo Fisher Scientific

Independent Ethics Committees/Institutional Review Boards consulted

Country	Name and address of Independent Ethics Committee/Institutional Review Board
Argentina	Comite de Etica Independiente Consultorios Integrados, Rosario, Santa Fe, Argentina, Italia 424, Rosario, Santa Fe S2000DEJ
Austria	Ethics Committee of the Medical University of Innsbruck, Medical University of Innsbruck, Anichstrasse 35, Innsbruck, Innsbruck A-6020
Belgium	Comité d'Ethique Erasme - ULB, Hospital<br>Facultaire Erasme-ULB, Route de Lennik 808, Brussels, Brussels 1070
China	Institute of Hematology and Blood Diseases Hospital Chinese Academy of Medical Sciences (Institute of Hematology, Chinese Academy of Medical Sciences) Ethics Review Committee, Institute of Hematology and Blood Diseases Hospital Chinese Academy of Medical Sciences (Institute of Hematology, Chinese Academy of Medical Sciences), 288 Nanjinglu, Heping District, Tianjin, China 300020
	Medical Ethics Committee of Zhongshan Hospital, Fudan University (Xiamen Branch), Zhongshan Hospital, Fudan University (Xiamen Branch), No. 668, Jinhu Road, Huli District, Xiamen, Fujian Province, China 361015
	Medical Ethics Committee of Henan Cancer Hospital, Henan Cancer Hospital, 127 Dongming Road, Jinshui District, Zhengzhou, Henan Province, China 450003
	Ethic Committee on Clinical Trial, West China Hospital of Sichuan University, No.37 Guoxue lane, Wuhou District, Chengdu, Sichuan Province, China 610041
Denmark	De Videnskabsetiske Medicinske Komitéer (VMK) Nationalt Center for Etik Enheden for Videnskab og Etik, Ørestads Boulevard 5, Bygning 35 + 37K, København S 2300
France	Comité de Protection des Personnes du Sud-Ouest et Outre-Mer 4, Cabanis Haut – Centre Hospitalier Esquirol, 15 rue du Docteur Raymond Marcland, BP 61730, Limoges Cedex 87025
Germany	Ethikkommission II der Universität Heidelberg (Med. Fakultät Mannheim), Haus 42, Ebene 3, Theodor-Kutzer-Ufer 1-3, Mannheim 68167
United Kingdom	West Midlands - Edgbaston Research Ethics Committee, 2 Redman Place, Stratford, London, London E20 1JQ
India	Institutional Ethics Committee Jawahar Lal Nehru Medical College, Kala Bagh, Ajmer, Rajasthan, India, Jahawar Lal Nehru Medical College, Kala Bagh, Ajmer, Rajasthan 305001
	Kaizen Ethics Committee, Kaizen Hospital, 132 Feet Ring Road, Near Helmet Circle, Memnagar, Ahmedabad 380052
	Institutional Ethics Committee, Vardhman Mahavir Medical College & Safdarjung Hospital, Ring Road Ansari Nagar, New Delhi, Delhi 110029
Israel	Kaplan Medical Center Helsinki Committee, Kaplan Medical Center, Pasternak Street, Rehovoth 76100
	IRB, Edith Wolfson Medical Center, 62 HaLohamim Street, Holon 5822012
	Meir Medical Center Helsinki Committee, Meir Medical Center, 59 Tchernichovsky Street, Kfar Saba 4428164
	Carmel Medical Center Helsinki Committee, Carmel Medical Center Helsinki, 7 Michal Street, Haifa 3436212

	Tel Aviv Sourasky MC Helsinki Committee, Tel Aviv Sourasky Medical Center, 6 Weizmann Street, Tel Aviv 64239
Italy	Comitato Etico Indipendente Di Area Vasta Emilia Centro via Albertoni 15, Bologna 40138
Japan	Tohoku University Hospital Institutional Review Board, Tohoku University Hospital, 1-1 Seiryo-machi, Aoba-ku, Sendai-shi, Miyagi 980-8574
	Kohnodai Hospital, National Center for Global Health and Medicine, National Center for Global Health and Medicine - Kohnodai Hospital, 1-7-1 Kohnodai, Ichikawa-shi, Chiba 272-8516
	Chiba Aoba Municipal Hospital Institutional Review Board, Chiba Aoba Municipal Hospital, 1273-2 Aobacho, Chuo-ku, Chiba-shi, Chiba 260-0852
	Kitano Hospital, Tazuke Kofukai Medical Research Institute Institutional Review Board, Kitano Hospital, Tazuke Kofukai Medical Research Institute, 2-4-20, Ohgimachi, Kita-ku, Osaka-shi, Osaka 530-8480
	Kanto Rosai Hospital Institutional Review Board, Kanto Rosai Hospital, 1-1 Kizukisumiyoshi-cho, Nakahara-ku, Kawasaki-shi, Kanagawa 211-8510
	Hyogo Medical University Hospital Institutional Review Board, Hyogo Medical University Hospital, 1-1 Mukogawa-cho, Nishinomiya-shi, Hyogo 663-8501
	Hamamatsu University Hospital Institutional Review Board, Hamamatsu University Hospital, 1-20-1 Handayama, Higashi-ku, Hamamatsu-shi, Shizuoka 431-3192
Netherlands	Medisch Ethische Toetsings Commissie Erasmus MC, Erasmus MC, Dr. Molewaterplein 40, Kamer Ae-337, Rotterdam, Zuid-Holland 3015 GD
Poland	Naczelna Komisja Bioetyczna do spraw badań klinicznych / Supreme Ethics
	Committee for Clinical Trials, Supreme Ethics Committee for Clinical Trials at Medical Research Agency, ul. Chmielna 69, Warszawa, Mazowieckie 00-801
South Korea	Asan Medical Center Institutional Review Board, Asan Medical Center, 88, Olympicro 43-gil, Songpa-gu, Seoul 05505
United States	WIRB, Emory University Hospital, 1019 39th Avenue SE, Suite 120, Puyallup, WA 98374-2115
	WIRB, National Institute of Allergy and Infectious Diseases, 1020 39th Avenue SE, Suite 121, Puyallup, WA 98374-2116
	Duke University Health System Institutional Review, Duke Allergy and Pulmonary South Durham, 2424 Erwin Rd., Hock Plaza, Ste 405, Durham, NC 27705
	WIRB, The Ohio State University Wexner Medical Center, 1020 39th Avenue SE, Suite 121, Puyallup, WA 98374-2116
	Human Research Protections Program, University of California San Diego, 9500 Gilman Drive, Mail Code 0052, La Jolla, CA 92093
	WIRB, Allergy Specialty Clinic and Food Allergy Clinic at Domino's Farms, 1019 39th Avenue SE, Suite 120, Puyallup, WA 98374-2115
	University of Utah IRB, University of Utah Health Care - Midvalley Health Center, 75 South 2000 East Research Administration Building, Salt Lake City, UT 84112
	WIRB, University Hospitals - Corporate, 1019 39th Avenue SE, Suite 120, Puyallup, WA 98374-2115

Supplementary methods

Rationale for benralizumab dose

The approved dose of benralizumab for severe eosinophilic asthma is 30 mg by subcutaneous (SC) injection every 4 weeks (Q4W) for the first three doses, followed by once every 8 weeks (Q8W) thereafter. Near-complete depletion of blood eosinophils has been observed with both the Q4W and Q8W regimens in both adults and adolescents.

Patients with hypereosinophilic syndrome (HES) generally have a greater blood and tissue eosinophil burden than patients with severe eosinophilic asthma. In an investigator-initiated phase 2 study of patients with varied clinical subtypes of HES, benralizumab 30 mg Q4W was effective in reducing blood and tissue eosinophil levels, whilst maintaining an acceptable safety profile. Given that depletion of eosinophils in the circulation as well as in the tissues of patients is considered important for the control of HES, the dosing regimen for benralizumab in this study was 30 mg SC Q4W for both adult and adolescent patients. The dosing for the phase 3 MANDARA study of benralizumab for eosinophilic granulomatosis with polyangiitis (EGPA) was also set at 30 mg Q4W from the results of the phase 2 HES study. Near-complete depletion of blood eosinophils was observed in the Phase 3 MANDARA study in patients receiving benralizumab 30 mg SC Q4W. This dose is now approved in the EGPA indication.

End-of-study definition

A patient was considered to have completed the study when they completed their last scheduled visit/telephone contact. The end of the study was defined as the last expected visit/contact of the last patient in the study. As patients were offered the opportunity to participate in an open-label extension (OLE) following completion of the double-blind treatment period, the end of the study was planned to be when the last randomized adult patient completes 1 year of OLE treatment and the last randomized adolescent patient completes 2 years of OLE treatment. However, the OLE treatment period could be extended or terminated early.

HES flare assessment

- Physical examinations:
  - A complete or brief physical examination was performed as part of the flare assessment
  - A complete physical examination included an assessment of the following: general appearance, respiratory, cardiovascular, abdomen, skin, head and neck (including ears, eyes, nose and throat), lymph nodes, thyroid, musculoskeletal (including spine and extremities), and neurological systems. A complete physical examination was conducted at screening and Visit 9 (Week 24)
  - A brief physical examination included an assessment of the following: general appearance, respiratory, cardiovascular, and abdomen. Brief physical examinations were conducted at all scheduled and flare visits
  - For flare visits, positive HES signs and symptoms were reported
  - The investigator used the HES physical examination to help identify specific organ involvement and to help identify subsequent flares during the study
- Investigator-led HES Symptom Interview:
  - The investigator-led HES symptom interview was completed by the investigator at screening and all scheduled and flare visits
  - The investigator asked questions from the investigator-led HES symptom interview to the patient and filled in the patient response using an electronic device
  - The investigator used the investigator-led HES symptom interview to document baseline HES symptoms and to help identify subsequent flares during the study
  - The investigator asked the patient if they were currently experiencing a specific symptom. If the patient answered ‘yes’, they were then asked to rate the intensity of the symptom (mild, moderate, or severe)
  - The symptoms were grouped into the following seven categories: general, skin, digestive, chest and breathing, nasal and vision, muscle and joint, and neurological
  - At screening (Visit 1), the investigator asked the patient to:
    - Choose and rank the top three most bothersome current symptoms (1 = most bothersome; 3 = least bothersome)
    - Rate the intensity of the symptom (no symptom, mild, moderate, or severe) when they were not experiencing a flare
- Laboratory Assessments:

    - Investigators reviewed laboratory abnormalities and other routine safety assessments (excluding the laboratory assessments that were to remain blinded until Week 28/Visit 10) to help identify a HES flare

Sensitivity analysis of the primary endpoint

A sensitivity analysis was performed on the primary analysis to assess the impact of changes to the patients' systemic background therapy before a flare. In this sensitivity analysis:

- Patients with an increase in systemic background therapy, or an increase in concomitant systemic therapy for another condition considered likely to impact the chance of a patient experiencing a flare, and did not experience a flare during the double-blind period were censored at the date of medication increase
- Patients with a decrease in systemic background therapy therapy considered likely to impact the chance of a patient experiencing a flare and prior to a flare and subsequently experienced a flare during the double-blind period were censored at the date of medication decrease
- All other patients will be handled as in the primary analysis

Statistical methods: Additional secondary endpoints and post hoc analyses

- Proportion of patients who experienced a HES flare during the DB period (key secondary endpoint)
    - The number of patients who had a first HES flare or withdraw prior to completing the DB period divided by the number of patients in the analysis set of interest
    - Patients who withdrew from the DB period early without having experienced a flare were considered as if they had experienced a flare event
- Number of HES worsening/flare events (rate/year) during the DB period (key secondary endpoint)
    - The number of times a patient had a HES flare during the DB period was counted. To be considered as a separate HES flare, the start date of a HES flare had to be at least 14 days after the stop date of the preceding HES flare. A HES flare that occurred within 14 days after the stop date of the preceding HES flare was combined and counted as a single event
    - Maximum follow-up time for calculation of the annualized flare rate was approximately 24 weeks. If a flare was ongoing at this date, the flare was counted in the calculation of annual flare rate, but the maximum follow-up time was truncated at the date described
    - The annual flare rate in each treatment group was calculated using: *Annual flare rate = 365.25 × total number of flares/total duration of follow-up within the treatment group (days)*
- Time to first hematologic relapse during the DB period (key secondary endpoint)
    - A first hematologic relapse was declared when AEC from central laboratory assessments post-baseline was ≥1,000 cells/µL for the first time during the DB period
    - The time to first hematologic relapse was calculated as: *start date of relapse – date of randomization + 1*
- Improvement in fatigue (PROMIS Fatigue) (key secondary endpoint)
    - PROMIS Fatigue Short Form 7a consists of 7 questions designed to assess the severity of fatigue-related symptoms and the impact of the symptoms on daily activities over the prior 7 days. The responses to each question are based on a 5-point Likert scale ranging from 1 to 5
    - A standardized T-score was obtained by rescaling the raw total score into a standardized score using the score conversion table (available online at: https://www.healthmeasures.net/images/PROMIS/manuals/Scoring_Manuals_/PROMIS_Fatigue_Scoring_Manual.pdf), with a mean of 50 and a standard deviation of 10:

Raw score	T-score	Standard error
7	29.4	5.3
8	33.4	4.8
9	36.9	4.3
10	39.6	4.0
11	41.9	3.8
12	43.9	3.5
13	45.8	3.3
14	47.6	3.2
15	49.2	3.1
16	50.8	3.0
17	52.2	3.0

18	53.7	3.0
19	55.1	3.0
20	56.4	2.9
21	57.8	2.9
22	59.2	2.9
23	60.6	2.9
24	62.0	2.9
25	63.4	2.9
26	64.8	2.9
27	66.3	2.9
28	67.8	2.9
29	69.4	2.9
30	71.1	3.0
31	72.9	3.0
32	74.8	3.1
33	77.1	3.3
34	79.8	3.6
35	83.2	4.1

- Proportion of patients with hematologic relapse during the DB period
    - The number of patients who had a first hematologic relapse in the DB period divided by the number of patients in the analysis set
    - Patients who withdrew from the DB period early without having had a hematologic relapse were considered as if they had experienced a hematologic relapse event
- Proportion of patients with AEC <500 cells/µL for 24 weeks
    - Patients who had AEC <500 cells/µL, from all of their available central laboratory eosinophil results for the 24-week DB period were considered as maintaining remission throughout the DB period
    - Proportion of patients who maintained remission was defined as the number of patients who maintained AEC <500 cells/µL divided by the number of patients in the analysis set
    - All AEC assessments including repeated, scheduled/unscheduled, and flare visits during DB treatment period were taken into account
    - If a patient had more than one visit window with a missing AEC assessment, including patients with missing assessments due to withdrawing from the study, they were considered as not achieving maintenance of AEC <500 cells/µL
- Proportion of patients requiring an increase in corticosteroid dose from baseline during the DB period
    - A patient who required an increase from their baseline corticosteroid dose of any amount at any point during the DB period was assigned a value of 1 for corticosteroid increase, and a patient with no increase in corticosteroid dose was assigned a value of 0
    - This included increasing dose of systemic OCS for patients already on systemic OCS or starting any systemic corticosteroids (SCS)
    - Medical review of increased OCS or initiation of SCS was conducted prior to unblinding to confirm that the change in medication was for the disease under study or for another condition with symptoms that are closely related to the patient's HES
    - Proportion of patients requiring an increase in corticosteroid dose from baseline at any point in the DB treatment period was calculated as the number of patients who required an increase in the DB period divided by the number of patients in the analysis set
    - The number of times a patient required a corticosteroid increase/burst was counted. To be considered a separate incidence of corticosteroid increase/burst, the start date of the increase/burst must be at least 14 days after the start date of the previous increase/burst
    - Systemic corticosteroid dose was converted to prednisone equivalent dose (corticosteroid dose/[conversion factor/10]); please refer to **Supplementary Table S1 Systemic corticosteroid dose equivalences**
    - Cumulative oral corticosteroid dose given over the DB period was calculated using the cumulative prednisone equivalent dose from baseline to 24 weeks. All patients were included, including those who did not take corticosteroid at baseline and/or during the study (with a cumulative dose of 0 mg)
- Improvement in health-related quality of life (HRQoL)

- The SF-36v2 (acute recall) is a 36-item, self-report survey of functional health and well-being, with a 1-week recall period. Responses to 35 of the 36 items are used to compute an 8-domain profile of functional health and well-being scores. The remaining 'health transition' item asks patients to rate how their current state of health compared to their state of health 1 week ago, and is not used to calculate domain scores
- The 8-domain profile consists of the following subscales: physical functioning (PF), role limitations due to physical health (RP), bodily pain (BP), general health perceptions (GH), vitality (VT), social functioning (SF), role limitations due to emotional problems (RE), and mental health (MH). Psychometrically based physical and mental health component summary scores (PCS and MCS, respectively) are computed from subscale scores
- Changes from baseline in SF-36v2 scales and component scores were analyzed using a mixed model for repeated measures with treatment group, baseline score, visit, region, and treatment visit interaction as covariates

- Improvement in Patient Global Impression of Severity (PGI-S) and Patient Global Impression of Change (PGI-C)
    - The PGI-S is a single item designed to capture the patient's perception of overall symptom severity over the past week of the assessment using a 6-point categorical response scale (0 = 'no symptoms' to 5 = 'very severe symptoms')
    - The PGI-C is a single item assessment to evaluate the patient's perception of change in health status. The patient is asked to report the degree to which they have changed since entering the treatment period using a 7-point scale (1 = 'much better' to 7 = 'much worse')
    - For PGI-C, patients were also categorized according to the following improvement categories at post baseline:
        a. A little better, moderately better, much better → 'A little better'
        b. Moderately better, much better → 'Moderately better'
        c. Much better → 'Much better'
    - PGI-S and PGI-C responses, and PGI-C improvement categories were summarized with descriptive statistics by treatment group and visit
- Pharmacokinetic variables
    - Blood samples for pharmacokinetic assessments were collected from all patients. The baseline sample was collected prior to first investigational product administration. Serum concentrations for benralizumab-treated patients were determined using a validated electro-chemiluminescent immunoassay
    - The drug concentration levels were summarized using descriptive statistics at each visit for patients in the benralizumab treatment group. Results below the lower limit of quantification (LLOQ) were set to LLOQ/2 for analysis and listed as <LLOQ
    - The following cases were excluded from the pharmacokinetics (PK) analyses:
        - Benralizumab patients with all collected plasma concentration levels persistently below the lower limit of quantification (LLOQ = 7.72 ng/mL) throughout the DB treatment period
        - Timepoints from benralizumab patients where the collected plasma concentration was in excess of what is considered to be physiologically possible with dosing: ≥12,000 ng/mL for the benralizumab treatment group (only the timepoints with a result not physiologically possible were excluded)
- Immunogenicity variables
    - The anti-drug antibody (ADA) response variables were summarized as counts and percentages across the DB period by treatment group for the safety analysis set
    - Patients with a missing baseline ADA assessment were assumed to be ADA-negative at baseline as a conservative approach to ensure that all subjects were included in all analyses
- Post hoc subgroup analyses were conducted for the primary endpoint according to baseline organ involvement defined as a Yes/No classification for each organ system (pulmonary, dermatologic, gastrointestinal, musculoskeletal, or sinus). The Cox proportional hazards model included treatment group, organ involvement subgroup, and the interaction between the two as covariates.

Supplementary safety results

Narrative of fatality reported in this study

One elderly patient with a medical history of HES flares, had an adverse event with an outcome of death during the DB period in the benralizumab group. Thirty days after the second dose of benralizumab, the patient was hospitalized with ketoacidosis, COVID-19 and a suspicion of infectious enteritis. Two days later the patient died from sepsis. The cause of the sepsis was unknown as blood cultures were negative. The patient was taking prednisolone as a concomitant medication. The event was assessed as not possibly related to benralizumab treatment by the investigator.

Supplementary figures

Supplementary Fig. 1 | NATRON study design

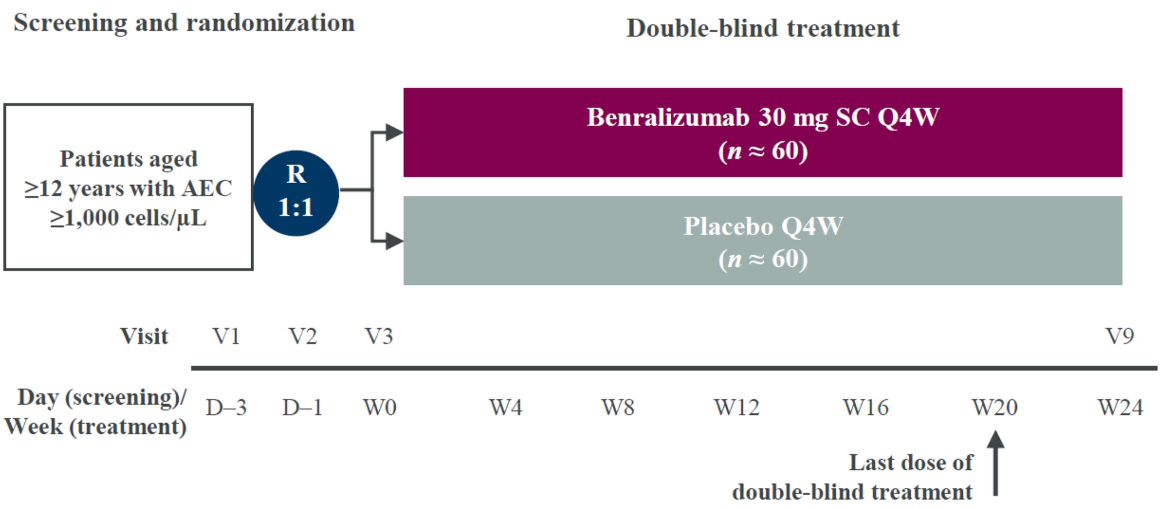

At Week 24, all patients were given the option to continue in the study and receive benralizumab 30 mg SC Q4W during an open-label extension period.

AEC, absolute eosinophil count; D, day; n, number of patients; Q4W, every 4 weeks; R, randomized; SC, subcutaneous; V, visit. W, week.

Supplementary Fig. 2 | Time to first HES flare (sensitivity analysis: patients changing systemic background therapy before HES flare)

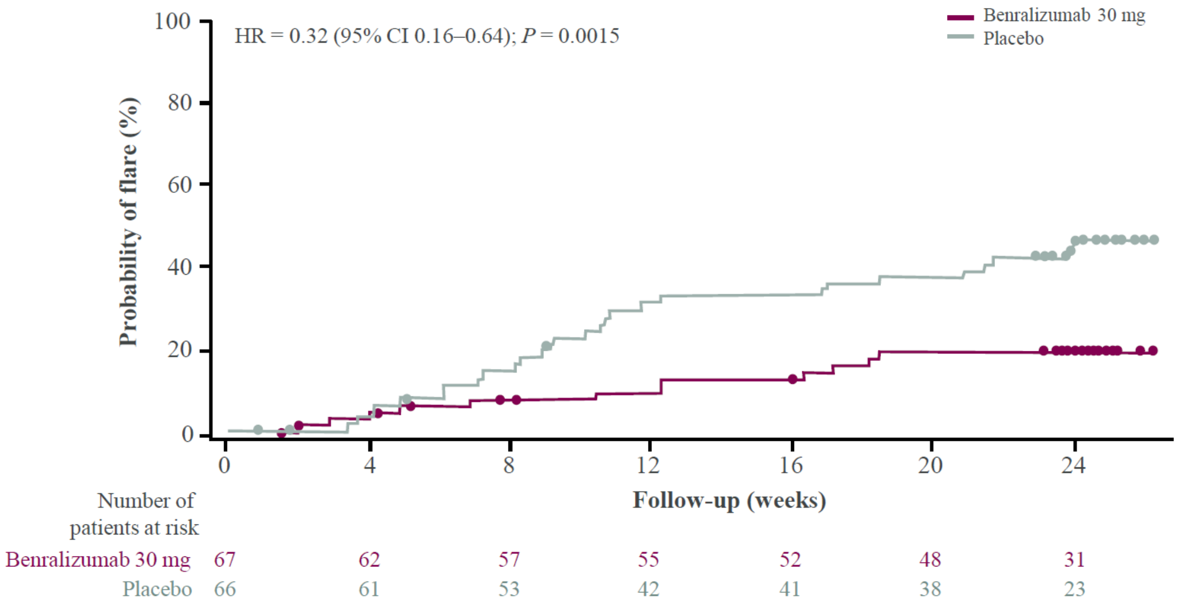

HR and 95% CIs were estimated using Cox proportional hazards model adjusted for treatment group, and unadjusted analysis was performed using an unstratified log-rank test

Patients with the intercurrent event of increase of background therapy or an increase in concomitant therapy for another condition considered likely to impact the chance of a patient flaring who did not have a flare in the double-blind period were censored at the date of background therapy increase. Patients with the intercurrent event of decrease of background therapy who flared during the double-blind period were censored at the date of background therapy decrease. Nine patients were censored: increased oral corticosteroids (4 benralizumab, 3 placebo); commenced dupilumab (1 placebo); stopped ciclosporin (1 benralizumab).

CI, confidence interval; HES, hypereosinophilic syndrome; HR, hazard ratio.

Supplementary Fig. 3 | Subgroup and post hoc analyses of time to first HES flare

a

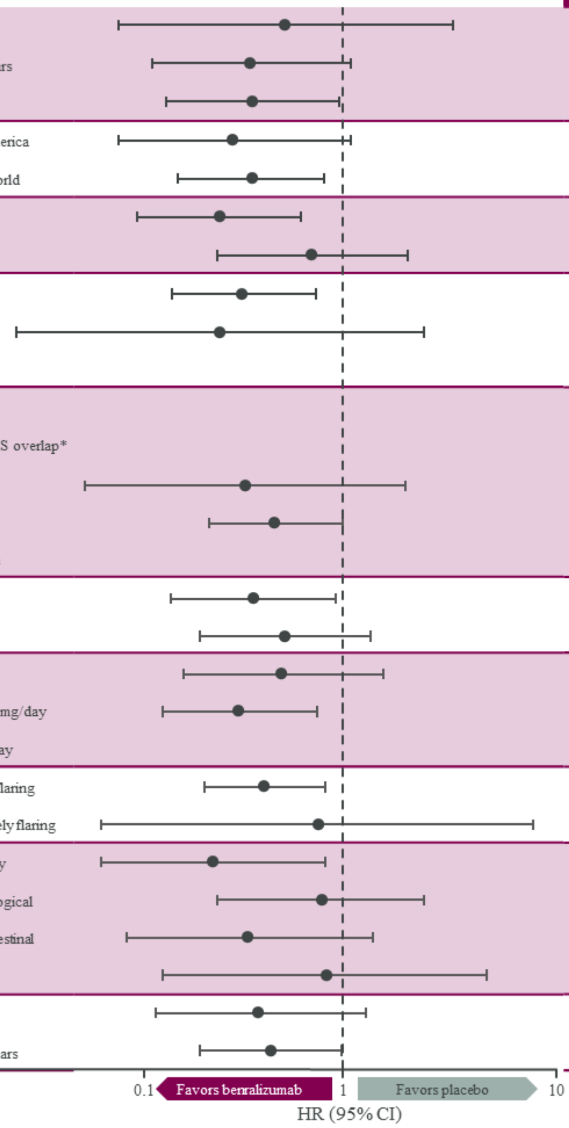

Subgroup		Benralizumab, *n/N* (%)	Placebo, *n/N* (%)	HR (95% CI)	*P* value
Age	≤21 years	2/9 (22.2%)	3/7 (42.9%)	0.54 (0.09–3.26)	0.505
	22–49 years	5/26 (19.2%)	10/23 (43.5%)	0.37 (0.13–1.09)	0.071
	≥50 years	6/32 (18.8%)	15/36 (41.7%)	0·38 (0.15–0.97)	0.043
Region	North America	4/12 (33.3%)	6/9 (66.7%)	0.31 (0.09–1.10)	0.070
	Rest of world	9/55 (16.4%)	22/57 (38.6%)	0.38 (0.17–0.82)	0.013
Sex	Female	7/43 (16.3%)	19/39 (48.7%)	0.27 (0.11–0.64)	0.003
	Male	6/24 (25.0%)	9/27 (33.3%)	0.72 (0.26–2.02)	0.532
Race	White	9/42 (21.4%)	22/45 (48.9%)	0.34 (0.16–0.75)	0.007
	Asian	1/10 (10.0%)	4/11 (36.4%)	0.27 (0.03–2.39)	0.237
	Other	2/6 (33.3%)	1/1 (100.0%)	–	–
HES subtype	L-HES	0/5 (0.0%)	4/11 (36.4%)	0.0 (0.0–NR)	0.992
	EGPA/HES overlap*	0/3 (0.0%)	0/0 (0.0%)	–	–
	SO-HES†	3/10 (30.0%)	2/3 (66.7%)	0.30 (0.05–1.79)	0.186
	I-HES	10/49 (20.4%)	22/51 (43.1%)	0.42 (0.20–0.89)	0.024
	Unknown	0 (0.0%)	0/1 (0.0%)	–	–
Baseline AEC‡	≤median	6/32 (18.8%)	17/34 (50.0%)	0.33 (0.13–0.83)	0.019
	>median	7/35 (20.0%)	11/31 (35.5%)	0.47 (0.18–1.22)	0.123
Baseline OCS	0 mg/day	6/20 (30.0%)	6/11 (54.5%)	0.45 (0.15–1.41)	0.173
	>0 to ≤10 mg/day	7/43 (16.3%)	21/45 (46.7%)	0.28 (0.12–0.67)	0.004
	>10 mg/day	0/4 (0.0%)	1/10 (10.0%)	0.0 (0.0–NR)	0.991
HES flare status at randomization	Actively flaring	12/62 (19.4%)	26/59 (44.1%)	0.37 (0.19–0.73)	0.005
	Not actively flaring	1/5 (20.0%)	2/7 (28.6%)	0.68 (0.06–7.47)	0.750
Primary organ involvement	Pulmonary	3/27 (11.1%)	12/27 (44.4%)	0.21 (0.06–0.73)	0.015
	Dermatological	5/16 (31.3%)	7/17 (41.2%)	0.71 (0.22–2.22)	0.551
	Gastrointestinal	3/16 (18.8%)	6/12 (50.0%)	0.31 (0.08–1.25)	0.101
	Other	2/8 (25.0%)	3/10 (30.0%)	0.75 (0.12–4.46)	0.747
Time since HES diagnosis	≥5 years	4/17 (23.5%)	8/16 (50.0%)	0.35 (0.11–1.16)	0.086
	0 to <5 years	9/50 (18.0%)	20/50 (40.0%)	0.4 (0.18–0.88)	0.023

b

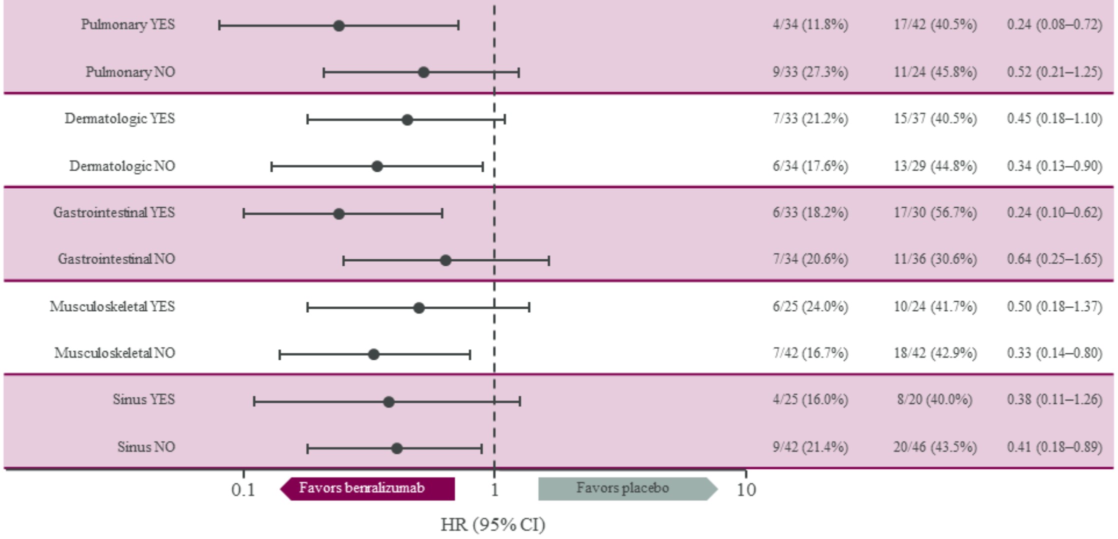

	Benralizumab, *n/N* (%)	Placebo, *n/N* (%)	HR (95% CI)
Pulmonary YES	4/34 (11.8%)	17/42 (40.5%)	0.24 (0.08–0.72)
Pulmonary NO	9/33 (27.3%)	11/24 (45.8%)	0.52 (0.21–1.25)
Dermatologic YES	7/33 (21.2%)	15/37 (40.5%)	0.45 (0.18–1.10)
Dermatologic NO	6/34 (17.6%)	13/29 (44.8%)	0.34 (0.13–0.90)
Gastrointestinal YES	6/33 (18.2%)	17/30 (56.7%)	0.24 (0.10–0.62)
Gastrointestinal NO	7/34 (20.6%)	11/36 (30.6%)	0.64 (0.25–1.65)
Musculoskeletal YES	6/25 (24.0%)	10/24 (41.7%)	0.50 (0.18–1.37)
Musculoskeletal NO	7/42 (16.7%)	18/42 (42.9%)	0.33 (0.14–0.80)
Sinus YES	4/25 (16.0%)	8/20 (40.0%)	0.38 (0.11–1.26)
Sinus NO	9/42 (21.4%)	20/46 (43.5%)	0.41 (0.18–0.89)

(**a**) Subgroup analysis of time to first HES flare. Cox proportional hazards models including treatment, subgroup, and treatment-by-subgroup interaction terms were used to estimate HRs and two-sided 95% CIs for each subgroup.
(**b**) Post-hoc analysis of time to first HES flare by the presence of any involvement of different organs (yes vs no). Cox proportional hazards model included treatment group, organ involvement subgroup, and the interaction between the two as covariates.

n numbers were too small to calculate HRs and CIs for the following subgroups: other race, L-HES, EGPA/HES overlap, HES subtype unknown, >10 mg/day baseline OCS. The time to first HES worsening/flare was calculated as start date of the first HES worsening – date of randomization + 1. For patients who did not experience a HES worsening/flare, the time to first HES worsening/flare was right censored at the end of the double-blind period corresponding to the earliest date of: the first of benralizumab open label dose, study Day 183, date of last contact, and data cut-off date. Rest of world includes Europe, Asia, and Argentina.

*HES with clinical features suggestive of EGPA (i.e., asthma, chronic rhinosinusitis with nasal polyposis), but ANCA-negative and no history of documented or suspected vasculitis.

†HES with involvement of a single organ system.

‡Median baseline (post-OCS responsiveness assessment) AEC was 115 cells/µL.

AEC, absolute eosinophil count; CI, confidence interval; EGPA, eosinophilic granulomatosis with polyangiitis; HES, hypereosinophilic syndrome; HR, hazard ratio; I-HES, idiopathic HES; L-HES, lymphocytic HES; N, number of patients in subgroup analysis; n, number of patients with events; NR, not reached; OCS, oral corticosteroids; SO-HES, single-organ HES.

Supplementary Fig. 4 | Blood eosinophil counts over the double-blind period

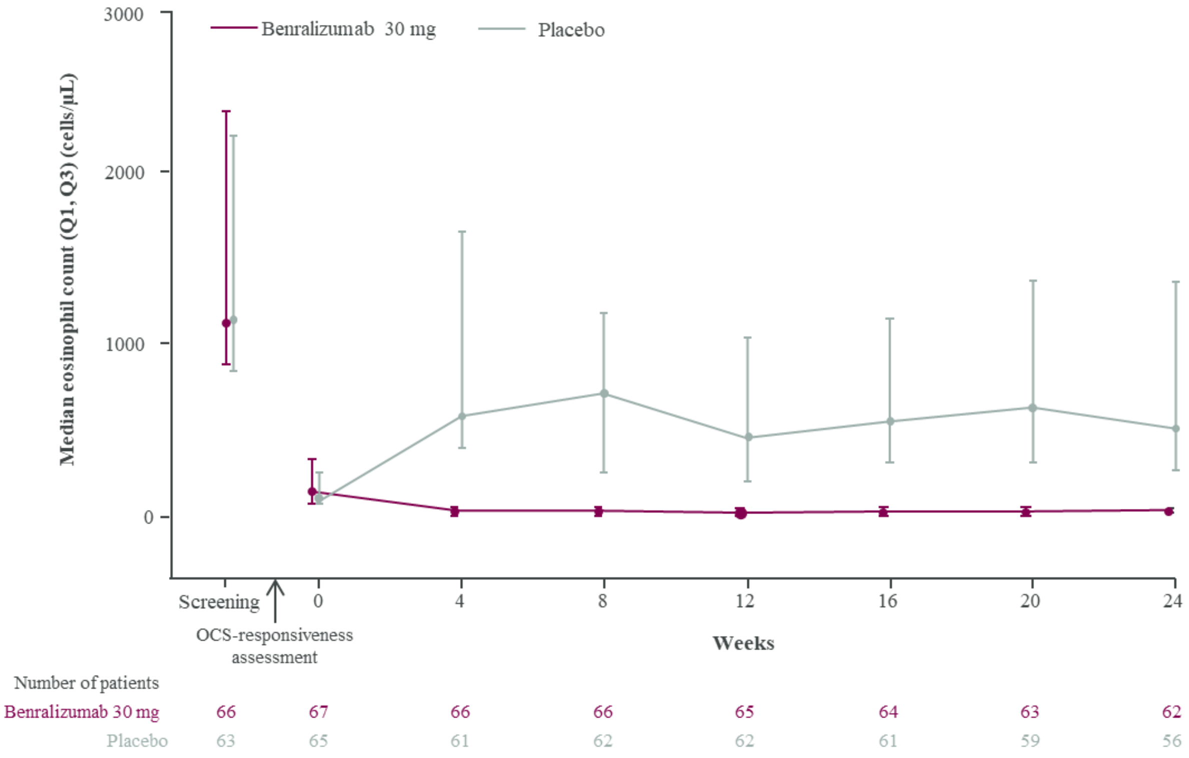

Error bars represent upper and lower quartiles. Screening visit was prior to OCS responsiveness assessment. All patients had a local laboratory eosinophil count above 1,000 cells/µL during screening and prior to corticosteroid-responsiveness test and randomization, which confirmed their eligibility for study participation. The above plot presents central laboratory results for eosinophil values and so some patients have a value <1,000 cells/µL at screening.

OCS, oral corticosteroid; Q1, lower quartile; Q3, upper quartile.

Supplementary Fig. 5 | Proportion of patients with hematologic relapse during the double-blind period

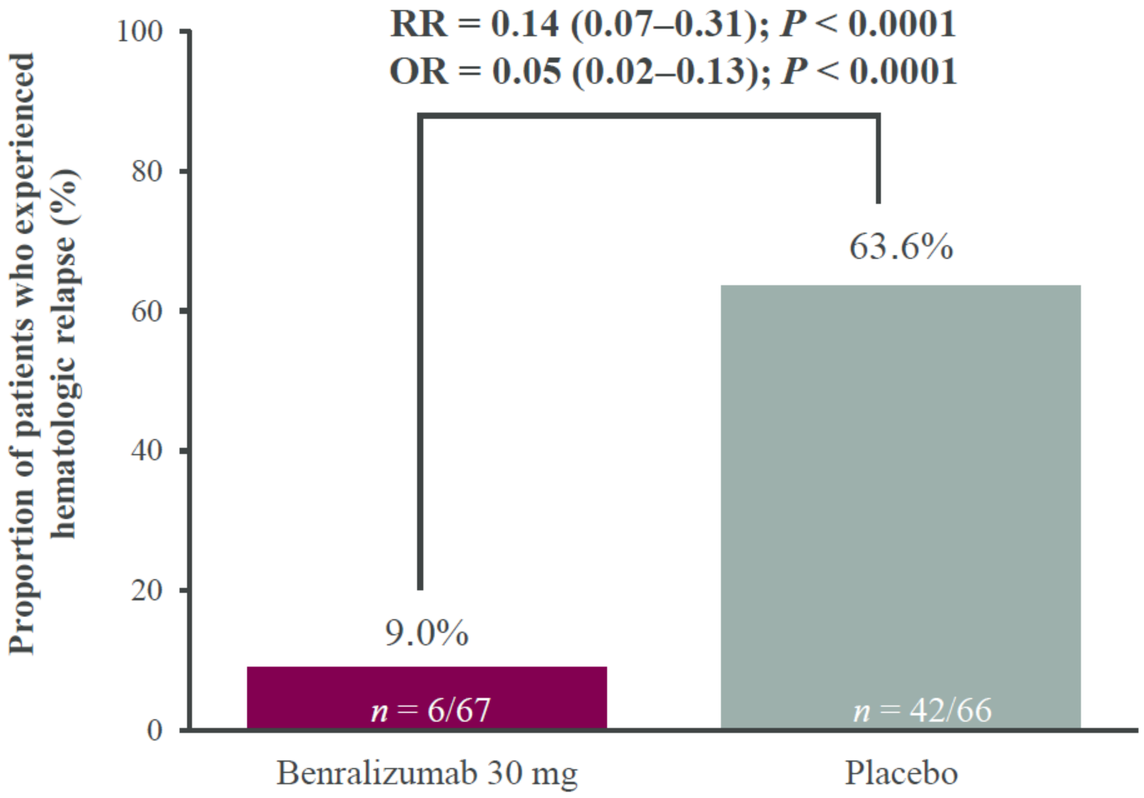

The proportion of patients who had a hematologic relapse or withdrew from the study over the DB period was defined as the number of patients who had a first hematologic relapse or withdrew prior to completing in the DB period divided by the number of patients in the analysis. Hematologic relapse was defined when AEC post baseline was ≥1,000 cells/μL for the first time. 95% CI was based on Mantel-Haenszel weight adjusted by region. OR, its 95% CI, and the associated *P* value were estimated using a logistic regression model including covariates for treatment and region. OR <1 favors benralizumab.

AEC, absolute eosinophil count; CI, confidence interval; DB, double-blind; n, number of patients with events; N, number of patients; OR, odds ratio; RR, relative risk.

Supplementary Fig. 6 | Proportion of patients with sustained AEC <500 cells/µL for 24 weeks

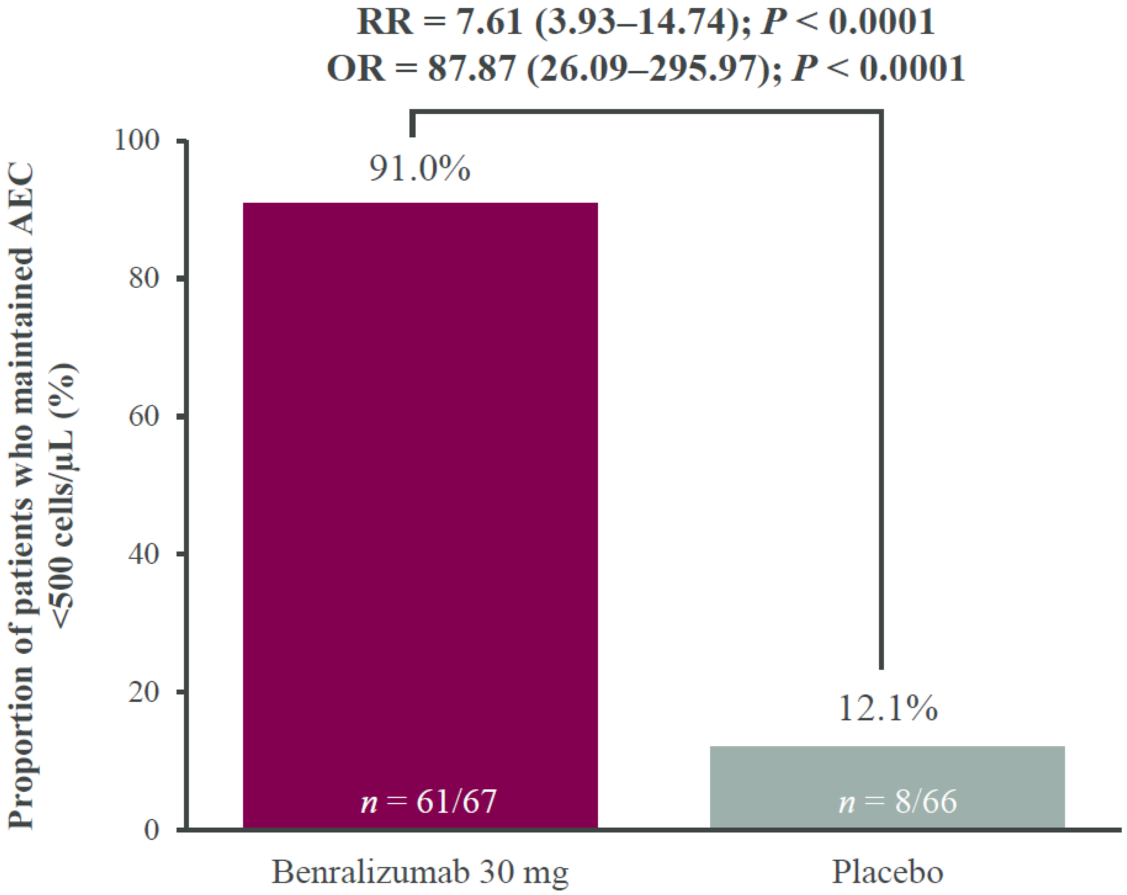

The proportion of patients who maintained AEC <500 cells/µL over the double-blind period was defined as the number of patients who maintained AEC <500 cells/µL divided by the number of patients in the analysis set. 95% CI was based on Mantel-Haenszel weight adjusted by region. OR, 95% CI, and the associated *P* value were estimated using a logistic regression model including covariates for treatment and region. OR >1 favors benralizumab.

Patients were receiving background therapy throughout the double-blind period

AEC, absolute eosinophil count; CI, confidence interval; N, number of patients; OR, odds ratio; RR, relative risk

Supplementary Fig. 7 | Systemic corticosteroid use during the double-blind period*

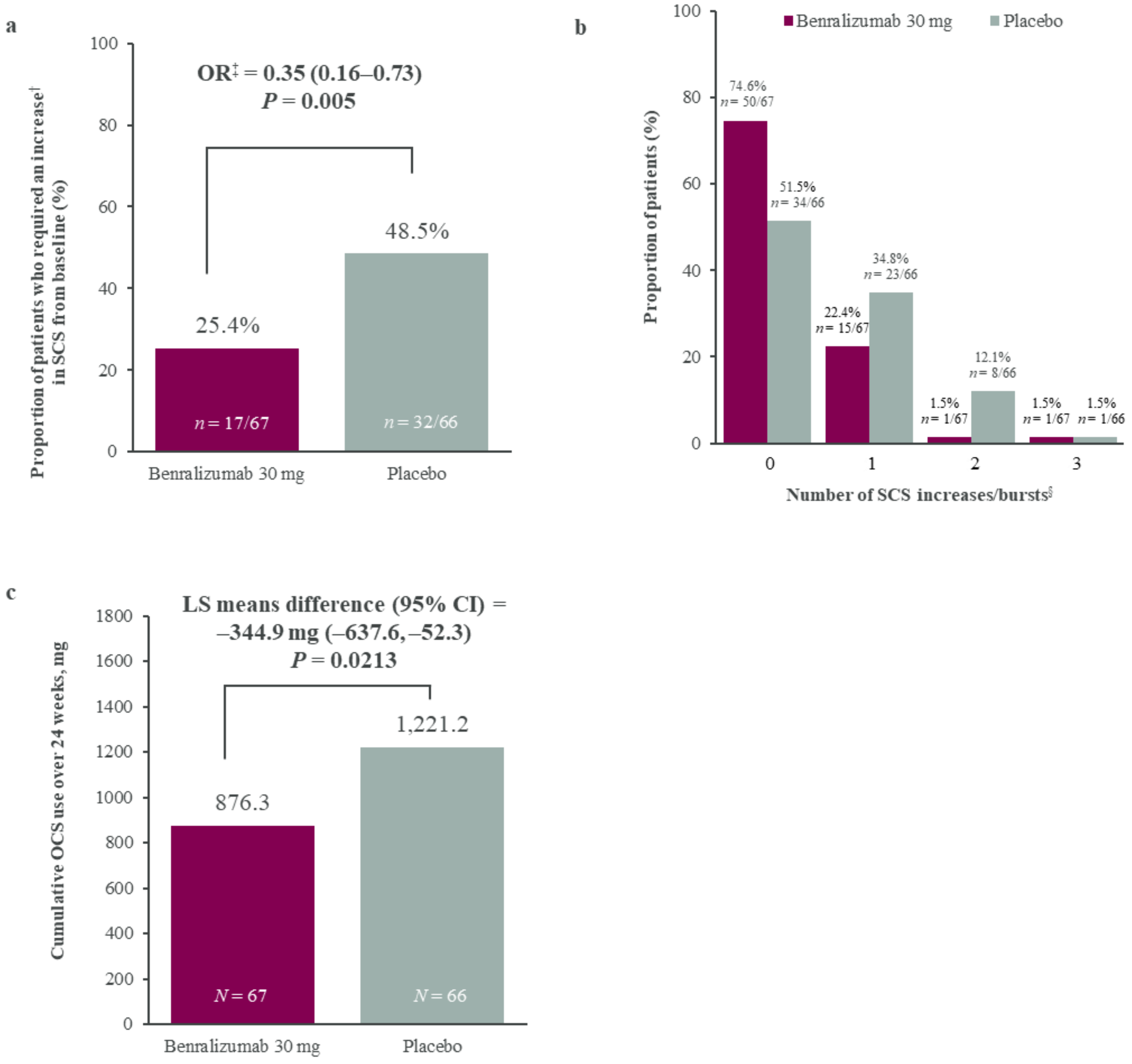

(**a**) Proportion of patients who required an increase in SCS from baseline.
(**b**) Proportion of patients with multiple SCS bursts.
(**c**) Cumulative OCS use over 24 weeks.

The number of patients with corticosteroid dose increases did not align with the number of patients with flares due to two patients receiving <10 mg prednisone equivalent OCS increases, which did not meet the flare definition.

SCS may have included the use of intravenous corticosteroids.

*Double-blind period started from the date of randomization until the earliest date of: date of the first of benralizumab open label dose, study Day 183, date of last contact, and data cut-off date.

†Included any increase of at least 1 mg prednisone equivalent versus the previous day where reason for therapy was HES flare or for another condition with closely related symptoms.

[‡]OR and its 95% CI were estimated using a logistic regression model including covariates for treatment and region. A corresponding *P* value was estimated using the Cochran-Mantel-Haenszel method adjusted by region. ORs lower than 1 are in favor of benralizumab effect.

[§]A single systemic corticosteroid increase/burst was considered if the start date of a systemic corticosteroid increase/burst was at least 14 days after the start date of the previous increase/burst.

CI, confidence interval; HES, hypereosinophilic syndrome; LS, least squares; n/N, number of patients; OCS, oral corticosteroids; OR, odds ratio; SCS, systemic corticosteroids.

Supplementary Fig. 8 | LS mean change from baseline in SF-36v2 component scores

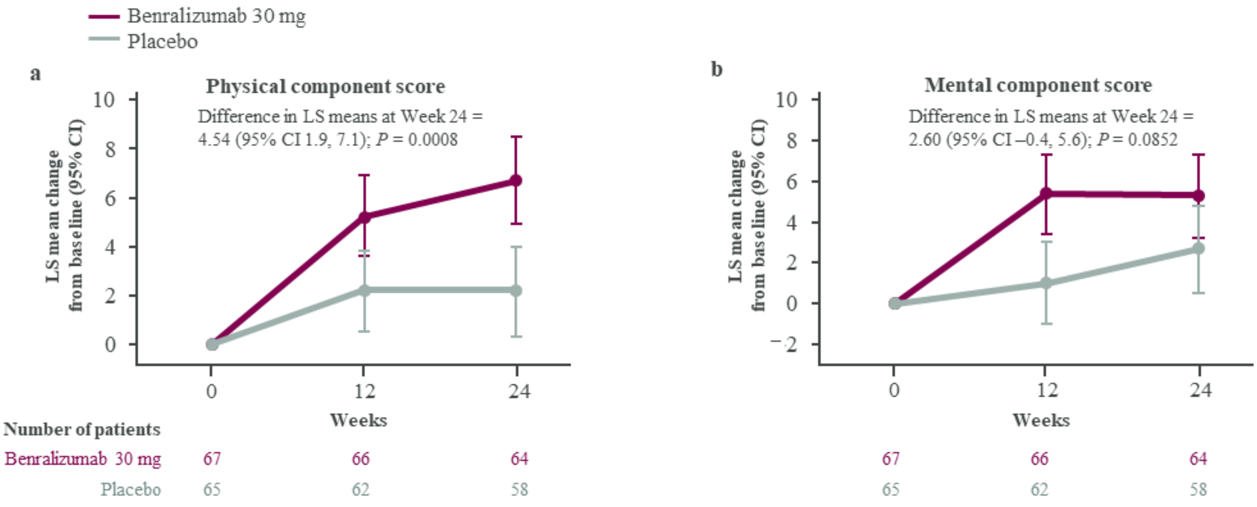

(**a**) LS mean change from baseline in SF-36v2 physical component score.
(**b**) LS mean change from baseline in SF-36v2 mental component score.

An increase in score indicates an improvement. Error bars are 95% CIs. LS means difference was estimated using a repeated measures ANCOVA analysis.

CI, confidence interval; LS, least squares; SF-36v2, Short Form 36 Health Survey version 2.

Supplementary Fig. 9 | PGI-S category from baseline through to Week 24

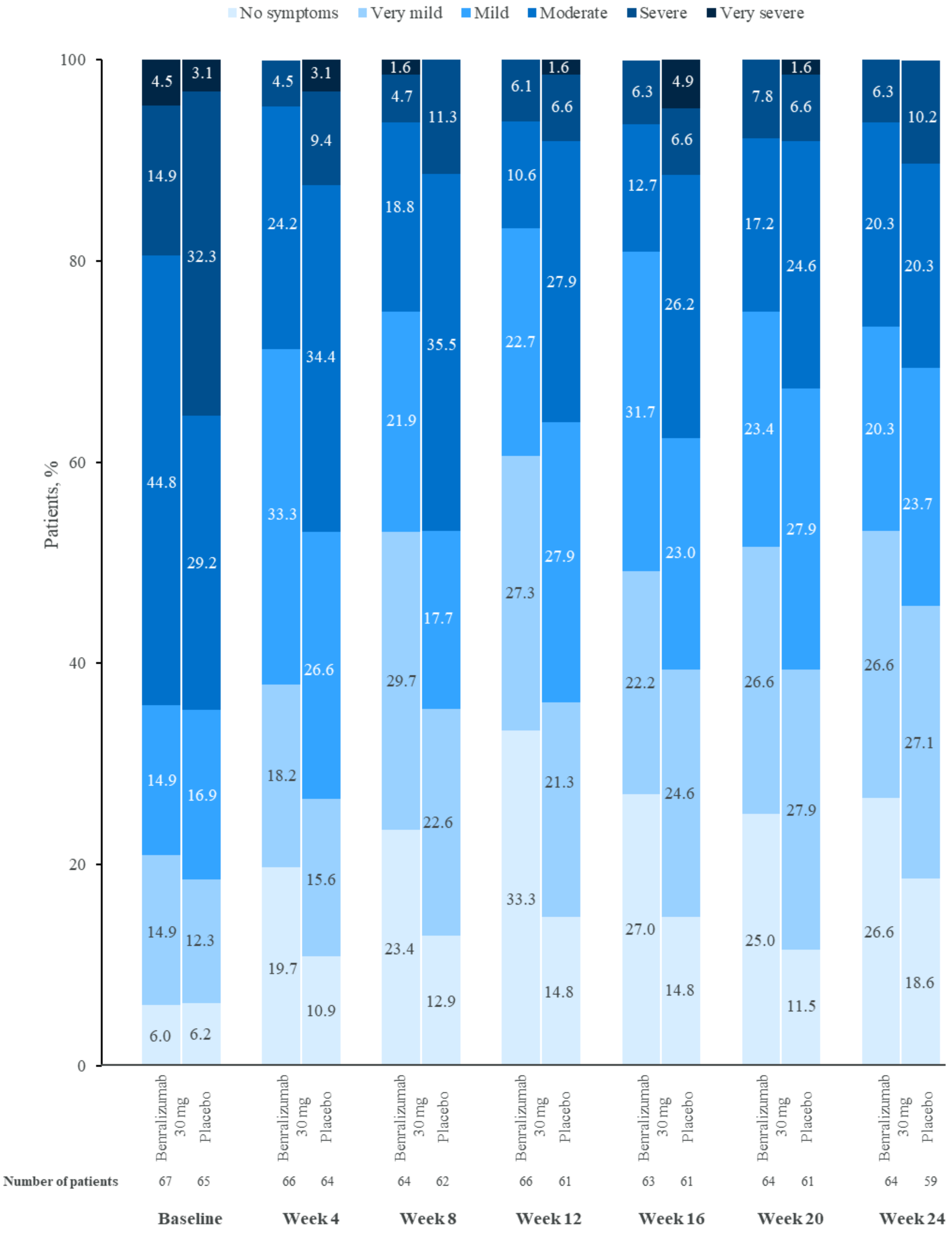

PGI-S is a single item to capture the patient's perception of overall symptom severity. Percentages used the total number of patients in each treatment group and visit with a completed assessment as the denominator. Baseline was defined as the last valid value on or prior to the date of randomization.

PGI-S, patient global impression of severity.

Supplementary Fig. 10 | PGI-C category from Week 4 through to Week 24

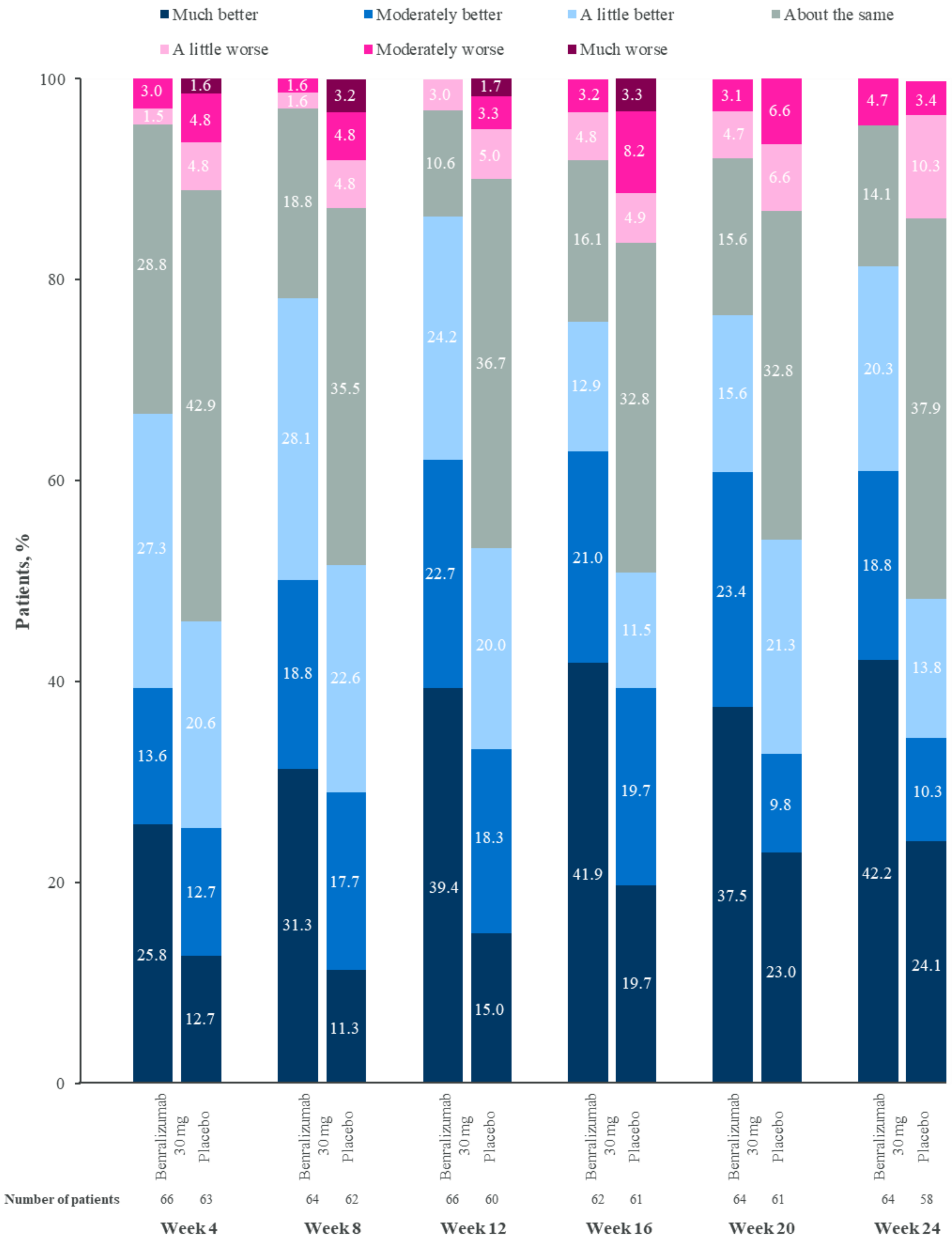

PGI-C is a single item to capture the patient's overall evaluation of response to treatment. Percentages used the total number of patients in each treatment group and visit with a completed assessment as the denominator.

PGI-C, patient global impression of change.

Supplementary Fig. 11 | Benralizumab serum concentrations (PK analysis set)

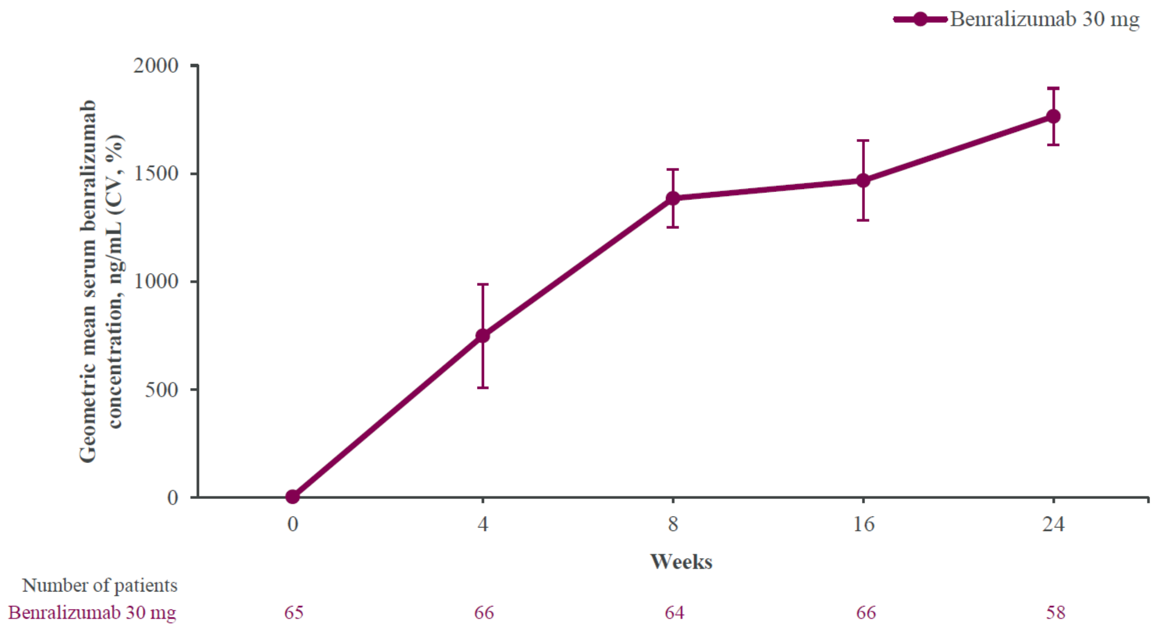

PK Serum samples were collected pre dose at each visit. The following cases were excluded from the PK analyses: benralizumab patients with all collected serum concentration levels persistently below the lower limit of quantification (LLOQ = 7.72 ng/mL, 3.86 ng/mL for Chinese patients) throughout the DB treatment period. Timepoints from benralizumab patients where the collected plasma concentration was in excess of what was considered to be physiologically possible with dosing: ≥12,000 ng/mL for the benralizumab treatment group (only the timepoints with a result not physiologically possible were excluded).

CV, coefficient of variation; DB, double-blind; LLOQ, lower limit of quantification; PK, pharmacokinetic.

Supplementary tables

Supplementary Table 1 | Systemic corticosteroid dose equivalences

Systemic corticosteroid	Approximate equivalence dose (mg)
Cortisone	50.0
Hydrocortisone	40.0
Deflazacort	12.0
Prednisolone	10.0
Prednisone	10.0
Methylprednisolone	8.0
Triamcinolone	8.0
Budesonide	4.2
Dexamethasone	1.5
Betamethasone	1.2

Supplementary Table 2 | Additional baseline characteristics

Characteristic	Benralizumab (*n* = 67)	Placebo (*n* = 66)	Total (*N* = 133)
**Age**			
Mean (SD)	47.5 (18.62)	49.2 (18.86)	48.4 (18.68)
**Ethnic group, *n* (%)**			
Hispanic or Latino	1 (1.5%)	1 (1.5%)	2 (1.5%)
Not Hispanic or Latino	66 (98.5%)	65 (98.5%)	131 (98.5%)
**Height (cm)**			
Mean (SD)	167.9 (10.03)	168.2 (9.46)	168.0 (9.71)
Median (range)	166.0 (149.0–193.0)	166.4 (148.0–191.0)	166.0 (148.0–193.0)
**Weight (kg)**			
Mean (SD)	70.9 (16.95)	74.8 (22.88)	72.8 (20.13)
Median (range)	70.0 (40.0–118.3)	69.0 (46.0–147.0)	70.0 (40.0–147.0)
**BMI (kg/m^2)**			
Mean (SD)	25.0 (4.54)	26.2 (6.43)	25.6 (5.57)
Median (range)	24.1 (16.6–38.6)	24.2 (17.3–47.4)	24.1 (16.6–47.4)
**HES primary organ involvement, *n* (%)[a]**			
Pulmonary	27 (40.3%)	27 (40.9%)	54 (40.6%)
Dermatologic	16 (23.9%)	17 (25.8%)	33 (24.8%)
Gastrointestinal	16 (23.9%)	12 (18.2%)	28 (21.1%)
Musculoskeletal	3 (4.5%)	5 (7.6%)	8 (6.0%)
Sinus	1 (1.5%)	3 (4.5%)	4 (3.0%)
Cardiac	1 (1.5%)	0	1 (0.8%)
Neurological	1 (1.5%)	0	1 (0.8%)
Other	2 (3.0%)	2 (3.0)	4 (3.0%)
**Median (range) peak absolute eosinophil count in previous 12 months (cells/µL)**	3,115.0 (1,100.0–51,690.0)	2,689.3 (274.9–27,200.0)	2,915.0 (274.9–51,690.0)
**Eosinophil count at screening – central result (cells/µL)[b]**			
Geometric mean	1,244.0	1,326.4	1,283.6
Median (range)	1,085.0 (360–5,620)	1,120.0 (60–20,910)	1,100.0 (60–20,910)
**Prior HES-related biologic therapy in the past 12 months, *n* (%)[c]**			
Benralizumab	0	2 (3.0%)	2 (1.5%)
Dupilumab	1 (1.5%)	1 (1.5%)	2 (1.5%)
Mepolizumab	5 (7.5%)	4 (6.1%)	9 (6.8%)
Omalizumab	2 (3.0%)	0	2 (1.5%)
Rituximab	2 (3.0%)	0	2 (1.5%)
Vedolizumab	0	1 (1.5%)	1 (0.8%)
**Background corticosteroid medication types, *n* (%)**			
Any topical corticosteroid	32 (47.8%)	37 (56.1%)	69 (51.9%)
Oral locally acting corticosteroids	3 (4.5%)	1 (1.5%)	4 (3.0%)
Inhaled corticosteroids	25 (37.3%)	30 (45.5%)	55 (41.4%)
Single therapies	8 (11.9%)	10 (15.2%)	18 (13.5%)
ICS/LABA	22 (32.8%)	25 (37.9%)	47 (35.3%)
Triple therapies	2 (3.0%)	1 (1.5%)	3 (2.3%)
Topical nasal corticosteroids	9 (13.4%)	10 (15.2%)	19 (14.3%)
Dermatologic preparations of corticosteroids	2 (3.0%)	5 (7.6%)	7 (5.3%)
Ophthalmological corticosteroids	0	0	0
**Background HES-related antineoplastic and immunomodulating therapy at baseline, *n* (%)[d]**			
**Antineoplastic agents**			
Hydroxycarbamide	2 (3.0%)	3 (4.5%)	5 (3.8%)
Imatinib	1 (1.5%)	0	1 (0.8%)
Imatinib mesilate	1 (1.5%)	0	1 (0.8%)
**Immunostimulants**			
Peginterferon	0	1 (1.5%)	1 (0.8%)
Peginterferon alpha-2a	1 (1.5%)	0	1 (0.8%)
**Immunosuppressants**			
Azathioprine	0	1 (1.5%)	1 (0.8%)
Ciclosporin	1 (1.5%)	0	1 (0.8%)
Mycophenolate mofetil	1 (1.5%)	0	1 (0.8%)

[a]Primary organ involvement was determined by the investigator.

[b]Eligibility was confirmed based on local laboratory results. All patients had a valid eosinophil count above 1,000 cells/µL at local testing prior to randomization.

[c]One patient discontinued benralizumab more than two years prior to randomization, with the reason given as "subject recovered". The other patient discontinued benralizumab 5 months prior to randomization, with the reason given as "financial". Of the two patients previously on dupilumab, one stopped due to lack of efficacy (randomized to benralizumab group). This patient had also discontinued omalizumab previously for the same reason. The other dupilumab-treated patient, randomized to the placebo group, stopped dupilumab due to side effects. Of the nine patients who were previously on mepolizumab, eight discontinued due to lack of efficacy, and one (randomized to the benralizumab group), stopped mepolizumab due to side effects. This patient had also discontinued omalizumab previously for the same reason.

[d]Includes therapy started on or prior to randomization, and ongoing after randomization. Patients may have been receiving more than one therapy.

BMI, body mass index; HES, hypereosinophilic syndrome; ICS, inhaled corticosteroids; LABA, long-acting beta-agonist; SD, standard deviation.

Supplementary Table 3 | Anti-drug antibody response to benralizumab

n/*N* (%)	Benralizumab (*N* = 67)	Placebo (*N* = 66)
ADA negative (negative at all visits, baseline and post baseline)	59/67 (88.1%)	57/66 (86.4%)
ADA positive at baseline and/or post baseline (prevalence)	8/67 (11.9%)	9/66 (13.6%)
ADA positive only at baseline	0	0
ADA positive at both baseline and ≥ one post baseline	3/66 (4.5%)	5/65 (7.7%)
Treatment-emergent ADA positive[a]	7/66 (10.6%)	8/65 (12.3%)
Treatment-induced ADA positive[b]	5/66 (7.6%)	4/65 (6.2%)
Treatment-boosted ADA positive[c]	2/66 (3.0%)	4/65 (6.2%)
Non-treatment-emergent ADA positive[d]	1/66 (1.5%)	1/65 (1.5%)
Persistently positive ADA[e]	3/66 (4.5%)	1/65 (1.5%)
Transiently positive ADA[f]	2/66 (3.0%)	3/65 (4.6%)
Treatment-emergent ADA-positive with maximum post-baseline titre > median of maximum post-baseline titre[g]	4/66 (6.1%)	3/65 (4.6%)
nAb prevalence[h]	3/67 (4.5%)	5/66 (7.6%)
nAb incidence[i]	2/66 (3.0%)	3/65 (4.6%)

ADA positive at baseline and/or post baseline and treatment emergent ADA positive in the placebo group can be observed as a result of prior exposure to biologics

[a]A positive post-baseline result and either treatment-induced ADA positive or treatment-boosted ADA positive.

[b]Treatment induced ADA positive was defined as ADA negative at baseline and at least one post-baseline ADA positive assessment.

[c]Treatment-boosted ADA positive was defined as ADA positive at baseline and the baseline titre was boosted by greater than the variability of the assay (i.e., ≥4-fold) at ≥1 post-baseline assessment.

[d]Non-treatment emergent ADA positive was defined as ADA positive but not fulling the conditions above for treatment-emergent ADA positive.

[e]Persistently positive ADA was defined as ADA negative at baseline and having ≥2 post-baseline ADA positive assessments (with ≥16 weeks between first and last positive) or positive at the last available post baseline assessment.

[f]Transiently positive ADA was defined as ADA negative at baseline and ≥1 post-baseline ADA positive assessment but not fulfilling the conditions of persistently positive.

[g]The median of maximum titre was calculated based on the maximum titre of each ADA positive patient (including both baseline and post-baseline measurements).

[h]nAb prevalence was defined as nAb positive at any visit including baseline and/or post-baseline.

[i]nAb incidence was defined as nAb negative at baseline (or ADA negative at baseline) and nAb positive at any post-baseline visit

ADA, anti-drug antibody; nAb, neutralizing antibody.

Supplementary Table 4 | Adverse events by preferred term

Adverse event	Benralizumab (N = 67)	Placebo (N = 66)
**Patients with any adverse event, n (%)**	**43 (64.2%)**	**44 (66.7%)**
Headache	11 (16.4%)	5 (7.6%)
Upper respiratory tract infection	5 (7.5%)	5 (7.6%)
COVID-19	4 (6.0%)	4 (6.1%)
Influenza-like illness	4 (6.0%)	0
Arthralgia	3 (4.5%)	5 (7.6%)
Back pain	3 (4.5%)	0
Cough	3 (4.5%)	3 (4.5%)
Nasopharyngitis	3 (4.5%)	4 (6.1%)
Nausea	3 (4.5%)	0
Rash	3 (4.5%)	0
Rhinitis allergic	3 (4.5%)	1 (1.5%)
Abdominal pain	2 (3.0%)	1 (1.5%)
Abdominal pain upper	2 (3.0%)	0
Alopecia	2 (3.0%)	2 (3.0%)
Blood creatine phosphokinase increased	2 (3.0%)	0
Diverticulitis	2 (3.0%)	0
Dysphagia	2 (3.0%)	0
Influenza	2 (3.0%)	2 (3.0%)
Insomnia	2 (3.0%)	0
Malaise	2 (3.0%)	0
Myalgia	2 (3.0%)	2 (3.0%)
Pyrexia	2 (3.0%)	2 (3.0%)
Rhinitis	2 (3.0%)	0
Sinusitis	2 (3.0%)	1 (1.5%)
Urticaria	2 (3.0%)	0
Abdominal distention	1 (1.5%)	1 (1.5%)
Acne	1 (1.5%)	1 (1.5%)
Acute sinusitis	1 (1.5%)	0
Addison's disease	1 (1.5%)	0
Blood glucose increased	1 (1.5%)	0
Blood pressure increased	1 (1.5%)	0
Blood thyroid stimulating hormone increased	1 (1.5%)	1 (1.5%)
Breast pain	1 (1.5%)	0
Breast swelling	1 (1.5%)	0
Bronchitis	1 (1.5%)	1 (1.5%)
Candida infection	1 (1.5%)	1 (1.5%)
Cataract	1 (1.5%)	0
Cerumen impaction	1 (1.5%)	0
Chronic eosinophilic leukemia	1 (1.5%)	0
Conjunctival hemorrhage	1 (1.5%)	0
Conjunctivitis allergic	1 (1.5%)	0
Dermatitis contact	1 (1.5%)	0
Dizziness	1 (1.5%)	1 (1.5%)
Drug eruption	1 (1.5%)	0
Drug hypersensitivity	1 (1.5%)	1 (1.5%)
Dysmenorrhea	1 (1.5%)	0
Dyspnea	1 (1.5%)	0
Early satiety	1 (1.5%)	0
Eczema	1 (1.5%)	0
Folliculitis	1 (1.5%)	0
Food poisoning	1 (1.5%)	0
Gastric infection	1 (1.5%)	0
Gastritis	1 (1.5%)	0
Gastroenteritis	1 (1.5%)	2 (3.0%)
Helicobacter gastritis	1 (1.5%)	0
Herpes zoster	1 (1.5%)	1 (1.5%)
Hordeolum	1 (1.5%)	1 (1.5%)
Hypereosinophilic syndrome	1 (1.5%)	1 (1.5%)
Hypersensitivity	1 (1.5%)	0
Hypoaesthesia	1 (1.5%)	0
Hypoferritinemia	1 (1.5%)	0
Impetigo	1 (1.5%)	0
Incarcerated inguinal hernia	1 (1.5%)	0
Injection site edema	1 (1.5%)	0
Injection site pain	1 (1.5%)	0

Supplementary Table 4 | Adverse events by preferred term (cont.)

Adverse event	Benralizumab (N = 67)	Placebo (N = 66)
Injection site reaction	1 (1.5%)	0
Intention tremor	1 (1.5%)	0
Intraocular pressure increased	1 (1.5%)	0
Keloid scar	1 (1.5%)	0
Large intestine polyp	1 (1.5%)	0
Leukopenia	1 (1.5%)	0
Nasal congestion	1 (1.5%)	0
Neuropathy peripheral	1 (1.5%)	0
Neutrophil count decreased	1 (1.5%)	0
Non-cardiac chest pain	1 (1.5%)	0
Edema peripheral	1 (1.5%)	2 (3.0%)
Oropharyngeal pain	1 (1.5%)	0
Otitis externa	1 (1.5%)	0
Pain in extremity	1 (1.5%)	1 (1.5%)
Parainfluenza virus infection	1 (1.5%)	0
Pharyngitis	1 (1.5%)	0
Photophobia	1 (1.5%)	0
Pityriasis rosea	1 (1.5%)	0
Sepsis	1 (1.5%)	0
Skin laceration	1 (1.5%)	0
Spinal pain	1 (1.5%)	0
Staphylococcal skin infection	1 (1.5%)	0
Syncope	1 (1.5%)	0
Tendonitis	1 (1.5%)	1 (1.5%)
Traumatic pain	1 (1.5%)	0
Type 2 diabetes mellitus	1 (1.5%)	0
Urinary tract infection	1 (1.5%)	0
Vertigo	1 (1.5%)	0
Viral upper respiratory tract infection	1 (1.5%)	2 (3.0%)
Vitamin D deficiency	1 (1.5%)	0
Vulvovaginal mycotic infection	1 (1.5%)	0
Anemia	0	3 (4.5%)
Cystitis	0	3 (4.5%)
Fatigue	0	3 (4.5%)
Rhinorrhea	0	3 (4.5%)
Hypertension	0	2 (3.0%)
Night sweats	0	2 (3.0%)
Peripheral swelling	0	2 (3.0%)
Spinal stenosis	0	2 (3.0%)
Abdominal pain lower	0	1 (1.5%)
Adrenal insufficiency	0	1 (1.5%)
Anorectal polyp	0	1 (1.5%)
Anxiety disorder	0	1 (1.5%)
Asthenia	0	1 (1.5%)
Benign prostatic hyperplasia	0	1 (1.5%)
Burning sensation	0	1 (1.5%)
Bursitis	0	1 (1.5%)
Cardiac failure	0	1 (1.5%)
Chalazion	0	1 (1.5%)
Chills	0	1 (1.5%)
Cholecystitis	0	1 (1.5%)
Conjunctivitis	0	1 (1.5%)
Constipation	0	1 (1.5%)
Cushingoid	0	1 (1.5%)
Dehydration	0	1 (1.5%)
Dental caries	0	1 (1.5%)
Dermatitis atopic	0	1 (1.5%)
Diarrhea	0	1 (1.5%)
Diplopia	0	1 (1.5%)
Dyspepsia	0	1 (1.5%)
Dysphonia	0	1 (1.5%)
Ear infection	0	1 (1.5%)
Epistaxis	0	1 (1.5%)
Erythema	0	1 (1.5%)
Fetal death	0	1 (1.5%)
Gamma-glutamyl transferase increased	0	1 (1.5%)

Supplementary Table 4 | Adverse events by preferred term (cont.)

Adverse event	Benralizumab (*N* = 67)	Placebo (*N* = 66)
Gastrointestinal infection	0	1 (1.5%)
Groin abscess	0	1 (1.5%)
Hematuria	0	1 (1.5%)
Hematuria traumatic	0	1 (1.5%)
Hemoglobin increased	0	1 (1.5%)
Hemorrhoids	0	1 (1.5%)
Heat stroke	0	1 (1.5%)
Hyperglycaemia	0	1 (1.5%)
Hypotension	0	1 (1.5%)
Joint stiffness	0	1 (1.5%)
Joint swelling	0	1 (1.5%)
Motion sickness	0	1 (1.5%)
Nystagmus	0	1 (1.5%)
Oral herpes	0	1 (1.5%)
Osteoarthritis	0	1 (1.5%)
Platelet count increased	0	1 (1.5%)
Pneumonia	0	1 (1.5%)
Pneumonia bacterial	0	1 (1.5%)
Polycythaemia	0	1 (1.5%)
Post inflammatory pigmentation change	0	1 (1.5%)
Post-acute COVID-19 syndrome	0	1 (1.5%)
Presyncope	0	1 (1.5%)
Procedural pain	0	1 (1.5%)
Purpura	0	1 (1.5%)
Rhabdomyolysis	0	1 (1.5%)
Rib fracture	0	1 (1.5%)
Sinus congestion	0	1 (1.5%)
Sleep disorder	0	1 (1.5%)
Swelling face	0	1 (1.5%)
Telangiectasia	0	1 (1.5%)
Thrombocytosis	0	1 (1.5%)
Tinnitus	0	1 (1.5%)
Tubulointerstitial nephritis	0	1 (1.5%)
Vaccination complication	0	1 (1.5%)
Vaginal discharge	0	1 (1.5%)
Vomiting	0	1 (1.5%)

MedDRA version 28.0.

COVID-19, coronavirus disease 2019; MedDRA, Medical Dictionary for Regulatory Activities.
