# Supplementary material for: Benralizumab versus placebo for hypereosinophilic syndrome: a randomized, placebo-controlled phase 3 trial
Source: Nat Med. 2026 Mar 31;32(6):2017–25. doi: 10.1038/s41591-026-04315-8 (PMC13278967; doi:10.1038/s41591-026-04315-8)
Supplement: Supplementary file 2 — Reporting Summary [file 41591_2026_4315_MOESM2_ESM.pdf]

nature portfolio

Corresponding author(s): Dr Amy Klion

Last updated by author(s): Jan 29, 2026

# Reporting Summary

Nature Portfolio wishes to improve the reproducibility of the work that we publish. This form provides structure for consistency and transparency in reporting. For further information on Nature Portfolio policies, see our Editorial Policies and the Editorial Policy Checklist.

## Statistics

For all statistical analyses, confirm that the following items are present in the figure legend, table legend, main text, or Methods section.

| n/a | Confirmed | |
|---|---|---|
| ☐ | ☒ | The exact sample size (*n*) for each experimental group/condition, given as a discrete number and unit of measurement |
| ☒ | ☐ | A statement on whether measurements were taken from distinct samples or whether the same sample was measured repeatedly |
| ☐ | ☒ | The statistical test(s) used AND whether they are one- or two-sided<br>*Only common tests should be described solely by name; describe more complex techniques in the Methods section.* |
| ☐ | ☒ | A description of all covariates tested |
| ☐ | ☒ | A description of any assumptions or corrections, such as tests of normality and adjustment for multiple comparisons |
| ☐ | ☒ | A full description of the statistical parameters including central tendency (e.g. means) or other basic estimates (e.g. regression coefficient) AND variation (e.g. standard deviation) or associated estimates of uncertainty (e.g. confidence intervals) |
| ☐ | ☒ | For null hypothesis testing, the test statistic (e.g. *F*, *t*, *r*) with confidence intervals, effect sizes, degrees of freedom and *P* value noted<br>*Give P values as exact values whenever suitable.* |
| ☒ | ☐ | For Bayesian analysis, information on the choice of priors and Markov chain Monte Carlo settings |
| ☐ | ☒ | For hierarchical and complex designs, identification of the appropriate level for tests and full reporting of outcomes |
| ☒ | ☐ | Estimates of effect sizes (e.g. Cohen's *d*, Pearson's *r*), indicating how they were calculated |

*Our web collection on statistics for biologists contains articles on many of the points above.*

## Software and code

Policy information about availability of computer code

| | |
|---|---|
| Data collection | Data were collected using electronic case report forms developed by AstraZeneca and completed by the investigators on behalf of the patients. |
| Data analysis | All data analyses were performed with SAS® System (SAS Institute Inc., Cary, NC) software. |

For manuscripts utilizing custom algorithms or software that are central to the research but not yet described in published literature, software must be made available to editors and reviewers. We strongly encourage code deposition in a community repository (e.g. GitHub). See the Nature Portfolio guidelines for submitting code & software for further information.

## Data

Policy information about availability of data

All manuscripts must include a data availability statement. This statement should provide the following information, where applicable:

- Accession codes, unique identifiers, or web links for publicly available datasets
- A description of any restrictions on data availability
- For clinical datasets or third party data, please ensure that the statement adheres to our policy

Data underlying the findings described in this manuscript can be requested in accordance with AstraZeneca's data sharing policy described online at https://astrazenecagrouptrials.pharmacm.com/ST/Submission/Disclosure.
Data for studies directly listed on Vivli can be requested through Vivli at https://www.vivli.org.

Data for studies not listed on Vivli can be requested through Vivli at https://vivli.org/members/enquiries-about-studies-not-listed-on-the-vivli-platform/. AstraZeneca Vivli member page is also available outlining further details: https://vivli.org/ourmember/astrazeneca/.

## Research involving human participants, their data, or biological material

Policy information about studies with human participants or human data. See also policy information about sex, gender (identity/presentation), and sexual orientation and race, ethnicity and racism.

| | |
|---|---|
| Reporting on sex and gender | The research findings from the NATRON primary results apply do not only apply to one sex or gender.<br>There were no inclusion or exclusion criteria for patients by sex or gender.<br>Biological sex was captured in electronic case report forms completed by investigators based on patient information.<br>Gender identity was not collected or analysed in this study.<br>An analysis of the primary outcome by sex was pre-specified as a subgroup analysis. |
| Reporting on race, ethnicity, or other socially relevant groupings | There were no inclusion or exclusion criteria for patients by race or ethnicity.<br>Race data was not collected for French study sites due to national legal restrictions.<br>Race and ethnicity data were captured in electronic case repost forms completed by the investigators based on patient-reported information.<br>Patients were asked:<br>- Do you consider yourself Hispanic/Latino or not Hispanic/Latino? (Response options: Yes / No)<br>- Which racial designation best describes you? (Response options: White / Black or African American / Asian / Other<br>- What was the 'Other' race? (Open-ended response. Responses provided included: Native Hawaiian or other Pacific islander, American Indian or Alaska native and Other).<br>An analysis of the primary outcome by race was pre-specified as a subgroup analysis. |
| Population characteristics | Demographics and baseline disease characteristics were generally balanced between treatment groups, and the NATRON study population was representative of the target HES population.<br>The profile of HES disease history was generally similar between groups and reflective of the protocol-intended patient population having HES. The use of concomitant background HES medications was similar between patients in the benralizumab and placebo groups. The majority of patients were receiving background HES therapy at baseline, with broadly similar proportions of patients in both groups receiving OCS and cytotoxic or immunosuppressive therapy. For patients receiving background OCS, the mean daily dose was similar between the treatment groups.<br>The primary endpoint was analysed with a stratified log-rank test adjusting for region and HES flare status at screening. Pre-specified subgroup analyses were conducted for the following factors: age, geographic region, HES flare status at screening, sex, HES subtype, race, baseline blood eosinophil count, baseline OCS daily dose, primary organ involvement and time since HES diagnosis.<br>The treatment effect observed for benralizumab over placebo in the overall population was consistent across all pre-defined subgroups. |
| Recruitment | Between 20 July 2020 and 13 November 2024, 158 patients with HES were enrolled with 134 patients randomized in 40 study centres across 15 countries (Argentina, Austria, Belgium, China, Denmark, France, Germany, India, Israel, Japan, The Netherlands, Poland, South Korea, United Kingdom, United States). 133 patients received ≥1 dose of treatment. Eligibility criteria and screening procedures minimized selection bias. All participants gave informed consent.<br>ICH E6 Good Clinical Practice guidelines were followed. Patients enrolled in the study were not compensated for their participation; however, reasonable reimbursement of expenses incurred by the patients (e.g. travel, parking) was provided if allowed by local regulations. This was stated clearly in the informed consent form. |
| Ethics oversight | Comite de Etica Independiente Consultorios Integrados, Argentina<br>Ethics Committee of the Medical University of Innsbruck, Austria<br>Comité d'Ethique Erasme - ULB, Hospital, Belgium<br>Institute of Hematology and Blood Diseases Hospital Chinese Academy of Medical Sciences (Institute of Hematology, Chinese Academy of Medical Sciences) Ethics Review Committee, China<br>Medical Ethics Committee of Zhongshan Hospital, Fudan University (Xiamen Branch), China<br>Medical Ethics Committee of Henan Cancer Hospital, China<br>Ethic Committee on Clinical Trial, West China Hospital of Sichuan University, China<br>De Videnskabsetiske Medicinske Komitéer (VMK) Nationalt Center for Etik Enheden for Videnskab og Etik, Denmark<br>Comite de Protection des Personnes du Sud-Ouest et Outre-Mer 4, Cabanis Haut – Centre Hospitalier Esquirol, France<br>Ethikkommission II der Universität Heidelberg (Med. Fakultät Mannheim), Germany<br>West Midlands - Edgbaston Research Ethics Committee, United Kingdom<br>Institutional Ethics Committee Jawahar Lal Nehru Medical College, India<br>Kaizen Ethics Committee, Kaizen Hospital, India<br>Institutional Ethics Committee, Vardhman Mahavir Medical College & Safdarjung Hospital, India<br>Kaplan Medical Center Helsinki Committee, Israel<br>IRB, Edith Wolfson Medical Center, Israel<br>Meir Medical Center Helsinki Committee, Israel<br>Carmel Medical Center Helsinki Committee, Israel<br>Tel Aviv Sourasky MC Helsinki Committee, Israel<br>Comitato Etico Indipendente Di Area Vasta Emilia Centro via Albertoni 15, Italy<br>Tohoku University Hospital Institutional Review Board, Japan<br>Kohnodai Hospital, National Center for Global Health and Medicine, Japan<br>Chiba Aoba Municipal Hospital Institutional Review Board, Japan<br>Kitano Hospital, Tazuke Kofukai Medical Research Institute Institutional Review Board, Japan<br>Kanto Rosai Hospital Institutional Review Board, Japan<br>Hyogo Medical University Hospital Institutional Review Board, Japan |

Hamamatsu University Hospital Institutional Review Board, Japan
Medisch Ethische Toetsings Commissie Erasmus MC, Netherlands
Naczelna Komisja Bioetyczna do spraw badań klinicznych / Supreme Ethics, Poland
Committee for Clinical Trials, Supreme Ethics Committee for Clinical Trials at Medical Research Agency, Poland
Asan Medical Center Institutional Review Board, South Korea
WIRB, Emory University Hospital, United States
WIRB, National Institute of Allergy and Infectious Diseases, United States
Duke University Health System Institutional Review, United States
WIRB, The Ohio State University Wexner Medical Center, United States
Human Research Protections Program, University of California San Diego, United States
WIRB, Allergy Specialty Clinic and Food Allergy Clinic at Domino's Farms, United States
University of Utah IRB, University of Utah Health Care, United States
WIRB, University Hospitals - Corporate, United States

Note that full information on the approval of the study protocol must also be provided in the manuscript.

# Field-specific reporting

Please select the one below that is the best fit for your research. If you are not sure, read the appropriate sections before making your selection.

☒ Life sciences ☐ Behavioural & social sciences ☐ Ecological, evolutionary & environmental sciences

For a reference copy of the document with all sections, see nature.com/documents/nr-reporting-summary-flat.pdf

# Life sciences study design

All studies must disclose on these points even when the disclosure is negative.

| | |
|---|---|
| Sample size | It was estimated that approximately 38 first HES flare events during the double-blind period were required to detect a statistically significant difference between treatment groups at the two-sided 5% significance level with approximately 80% power if the true treatment effect is a hazard ratio of 0.389 (equivalent to 30% of patients receiving benralizumab experiencing an event by the end of the double-blind period versus 60% of patients receiving placebo). Based on these assumptions, a sample size of approximately 120 was expected, although recruitment could continue beyond this to provide confidence that sufficient events would be observed once complete follow-up was achieved. |
| Data exclusions | All efficacy endpoints, demographics, and baseline characteristics were analyzed using the full analysis set, which included all randomized patients who received ≥1 dose of study treatment according to the intention-to-treat principle, irrespective of adherence to the protocol and continued trial participation. Pre-specified sensitivity analysis was conducted to assess the impact of patients changing systemic background therapy before HES flare, by censoring any patients with systemic OCS or immunosuppressive therapy changes that were considered to have a potential to impact the chance of the patient flaring. |
| Replication | Not applicable, this was a single phase 3 registrational clinical trial due to the rare nature of the disease under investigation. |
| Randomization | All patients were centrally assigned to a randomized study treatment using Interactive Web Response Systems (IWRS)/Interactive Voice Response Systems (IVRS). As patients became eligible for randomization, unique randomization codes were assigned sequentially in each stratum from a randomization list prepared by a computerized system provided on behalf of AstraZeneca. The randomization sequence was computer-generated centrally using a permuted block design of block size 4 and stratified by geographic region (North America, Europe, Asia, and Rest of World) and HES flare status at screening. |
| Blinding | Patients, sponsor, site staff, and investigators were blinded to treatment allocation and to patients' blood and biopsy leukocyte counts during the double-blind treatment period and up to Week 4 of the OLE. All packaging and labeling ensured blinding for all sponsor and investigational site staff. |

# Reporting for specific materials, systems and methods

We require information from authors about some types of materials, experimental systems and methods used in many studies. Here, indicate whether each material, system or method listed is relevant to your study. If you are not sure if a list item applies to your research, read the appropriate section before selecting a response.

## Materials & experimental systems

| n/a | Involved in the study |
|---|---|
| ☒ | ☐ Antibodies |
| ☒ | ☐ Eukaryotic cell lines |
| ☒ | ☐ Palaeontology and archaeology |
| ☒ | ☐ Animals and other organisms |
| ☐ | ☒ Clinical data |
| ☒ | ☐ Dual use research of concern |
| ☒ | ☐ Plants |

## Methods

| n/a | Involved in the study |
|---|---|
| ☒ | ☐ ChIP-seq |
| ☒ | ☐ Flow cytometry |
| ☒ | ☐ MRI-based neuroimaging |

# Clinical data

Policy information about clinical studies

All manuscripts should comply with the ICMJE guidelines for publication of clinical research and a completed CONSORT checklist must be included with all submissions.

**Clinical trial registration**

NCT04191304 ClinicalTrials.gov

**Study protocol**

Redacted protocol will be available as a supplementary file alongside the published article

**Data collection**

This study began on 22 July 2020 and the primary analysis data cut-off was 7 May 2025.
First subject enrolled: 20 July 2020
Last subject enrolled: 13 November 2024
Last subject last visit in double-blind period: 07 May 2025
This study was conducted at 40 sites across 15 countries (Argentina, Austria, Belgium, China, Denmark, France, Germany, India, Israel, Japan, the Netherlands, Poland, South Korea, the United Kingdom, and the United States). Data were collected using electronic case report forms developed by AstraZeneca and completed by the investigators at the study sites.

**Outcomes**

The primary endpoint was time to first HES flare during the 24-week, double-blind treatment period. A flare was defined as HES clinical manifestation or lab abnormality resulting in an increase of OCS ≥10 mg/day prednisone equivalent for ≥2 days, or an increase or addition of a new cytotoxic and/or immunosuppressive therapy, or hospitalization. Flares were assessed by the investigator through complete or brief physical examinations, an investigator-led HES symptom interview, laboratory assessments, and other routine safety assessments;. If patients were unable to attend the study site for flare assessment, medical records were collected and an investigator-led HES symptoms interview was recommended. Time to first HES flare was calculated as the number of days from the date of randomization to the start date of the first flare event, plus 1 day. The start date of HES flare was defined as the first day of increased dose/burst of OCS, first day of any increase or addition of new cytotoxic and/or immunosuppressive therapy, or date of hospital admission, whichever occurred first.

Secondary endpoints that were multiplicity-protected within the pre-specified statistical testing hierarchy were defined as 'key'. Key secondary endpoints were: (1) the proportion of patients with HES flares, with those who withdrew from the study without having experienced a flare considered as having had a flare event; (2) annualized rate of HES flares, assessed over a maximum follow-up period of 24 weeks or, for patients lost to follow-up, the follow-up time was defined as the duration from randomization to the last timepoint at which flare status could be evaluated, with distinct flares defined as those with onset occurring ≥14 days after the resolution of the previous flare; (3) time to first hematologic relapse (AEC ≥1,000 cells/µL), calculated as the number of days from the date of randomization to the start date of first hematologic relapse plus 1 day; and (4) change from baseline to Week 24 in PROMIS Fatigue, with a standardized total score calculated for each visit over the double-blind period.

Other secondary endpoints included the proportion of patients with hematologic relapse (including those who withdrew from the study) during the double-blind period, the proportion of patients with AEC <500 cells/µL for 24 weeks, the proportion of patients requiring an increase in corticosteroid dose at any time during the double-blind period, and other patient-reported outcomes (SF-36, PGI-S, and PGI-C).

# Plants

**Seed stocks**

N/A

**Novel plant genotypes**

N/A

**Authentication**

N/A
